# Estimating age‐dependent survival from age‐aggregated ringing data—extending the use of historical records

**DOI:** 10.1002/ece3.4820

**Published:** 2019-02-05

**Authors:** Marina Jiménez‐Muñoz, Diana J. Cole, Stephen N. Freeman, Robert A. Robinson, Stephen R. Baillie, Eleni Matechou

**Affiliations:** ^1^ School of Mathematics, Statistics and Actuarial Science University of Kent Canterbury UK; ^2^ Centre for Ecology & Hydrology Wallingford UK; ^3^ British Trust for Ornithology Thetford UK

**Keywords:** conditional model, identifiability, mark–recovery, parameter redundancy, tag recovery, *Thalasseus sandvicensis*, *Turdus merula*

## Abstract

Bird ring‐recovery data have been widely used to estimate demographic parameters such as survival probabilities since the mid‐20th century. However, while the total number of birds ringed each year is usually known, historical information on age at ringing is often not available. A standard ring‐recovery model, for which information on age at ringing is required, cannot be used when historical data are incomplete. We develop a new model to estimate age‐dependent survival probabilities from such historical data when age at ringing is not recorded; we call this the historical data model. This new model provides an extension to the model of Robinson, 2010, Ibis, 152, 651–795 by estimating the proportion of the ringed birds marked as juveniles as an additional parameter. We conduct a simulation study to examine the performance of the historical data model and compare it with other models including the standard and conditional ring‐recovery models. Simulation studies show that the approach of Robinson, 2010, Ibis, 152, 651–795 can cause bias in parameter estimates. In contrast, the historical data model yields similar parameter estimates to the standard model. Parameter redundancy results show that the newly developed historical data model is comparable to the standard ring‐recovery model, in terms of which parameters can be estimated, and has fewer identifiability issues than the conditional model. We illustrate the new proposed model using Blackbird and Sandwich Tern data. The new historical data model allows us to make full use of historical data and estimate the same parameters as the standard model with incomplete data, and in doing so, detect potential changes in demographic parameters further back in time.

## 1 INTRODUCTION

There is now a long history, and abundant literature, on the estimation of avian survival from ringed birds (see, e.g., Williams, Nichols, & Conroy, 2002). In Europe and North America, bird ringing activities started at the beginning of the 20th century. Data were then registered on paper, and many national schemes hold archives of such records, with extensive digital capture of data only beginning in the last two decades. The way in which such archival data were collected and stored means that the total number of fledged birds ringed in different age categories is difficult or often impossible to obtain, due to the sheer size and heterogeneity of the records, compromising our ability to understand historical variation in survival probabilities. This paper examines suitable methods for exploring such data.

While the mean annual survival probabilities of adult birds are generally assumed to stabilize beyond a certain age, those of younger birds are generally lower (Martin, 1995; Péron et al., 2016). The probability of a bird being recovered after death may also be age‐dependent, as behavior and habitat use vary with age. A rigorous model for birds of different ages must take such variation into account. This is not a problem when the numbers of birds ringed annually in the various age‐classes are known. If these age‐specific annual totals are known, then standard models such as those proposed by Brownie, Anderson, Burnham, and Robson (1985) and Freeman and Morgan (1992) can be used. If annual total numbers are unknown, it is possible to use a model that is conditional on the number of birds recovered. The most commonly used model assumes a constant probability of reporting after death for all members of the cohort (Seber, 1971), but this can result in biased parameter estimates (McCrea, Morgan, Brown, & Robinson, 2012).

Rather than a conditional model that ignores annual numbers ringed, Robinson (2010) describes a model for when the annual numbers of pulli (chicks) and of fledged birds are separately known, but the latter includes both fully mature, breeding birds and juvenile birds of the year, which will have very different survival prospects. Robinson (2010) proposes that such data may indeed be used to estimate survival, by assuming that a fixed proportion of the birds ringed as fledged birds were actually juveniles.

In this paper, we present a model where this proportion is an unknown parameter. We use general theory on parameter redundancy to show that this parameter can be estimated. Subsequently, we perform a simulation study to show that this model gives similar parameter estimates to the standard model that requires that all the ringing totals are known, and provide an analysis on Blackbirds *Turdus merula* and Sandwich Terns *Thalasseus sandvicensis* to show the relevance of the proposed model for different types of data.

## 2 MATERIALS AND METHODS

### 2.1 Data

The British Trust for Ornithology (BTO) has collected an extensive historical data set of the total number of birds ringed in Britain and Ireland since 1909. However, until 2000 the data were submitted by ringers in paper form. From 2010, there are digitized data distinguishing three age‐groups at ringing: pulli, juveniles, and adults. Before then, digitized data only contained two age categories for ringing totals: pulli, which are first‐year birds, and fledged birds, the latter meaning free‐flying birds of unknown age including juveniles (also first‐year birds) and adults. Therefore, we consider here that the first‐year birds can be ringed as either pulli or juveniles. Fully computerizing historical data or finding ringing totals manually for a particular species of interest is possible, but it is time‐consuming. This has been stated for several species.

To illustrate the methods described in this paper, we use simulated data and two example BTO data sets. The first data set is on Blackbirds for the years 1964–1983. These data are taken from Robinson, Baillie, and King (2012), can be found in Supporting Information Appendix S1 (Tables S1 and S2), and consist of birds ringed as adults, juveniles, and/or pulli birds during the breeding season (April–September). For this data set, the total numbers of birds ringed for each of the three age categories, pulli, juveniles, and adults, are separately known. This data set and the simulated data sets therefore allow comparison between existing methods that require known age‐specific totals at time of ringing and the methods developed in this paper for historical data. The second data set is on Sandwich Terns for the years 1970–1990. Sandwich Terns are summer visitors to Europe; thus, birds can only be ringed in the breeding season (June to August). For this data set, we only have information on the total number of birds ringed as pulli and fledged birds. The Sandwich Tern recovery data for adults and juveniles, presented in Supporting Information Appendix S2 (Tables S3–S5), are very sparse.

### 2.2 Models

In this section, we describe the models that can be fitted to different mark–recovery data sets. In all of the models, we suppose there are *n*_1_ years of ringing and *n*_2_ years of recovery of dead birds. The total number of birds ringed in age category *c*, in year *i*, is *T*_*c*,*i*_, and the number of birds ringed in age category *c*, in year *i* that were recovered dead in year *t*, is *N*_*c*,*i*,*t*_, for *i* = 1, …, *n*_1_, and *t* = *i*, …, *n*_2_. The age category *c* represents the age at which birds were ringed, which could be pulli, denoted by *p*, juveniles (*j*), first‐year birds (1), fledged birds (*f*), or adults (*a*). As mentioned in the data section, first‐year birds may be either pulli or juveniles, and fledged birds may be either juveniles or adults.

The models depend on the following parameters:

- *ϕ*_1,*t*_ is the annual survival probability for a first‐year bird alive at the start of year *t*;
- *ϕ*_*a*,*t*_ is the annual survival probability for an adult bird alive at the start of year *t*;
- *λ*_1,*t*_ is the annual probability of recovering a first‐year dead bird in year *t*;
- *λ*_*a*,*t*_ is the annual probability of recovering a dead adult bird in year *t*.

Note that we give the general form of each parameter above, but time dependence could be dropped in appropriate circumstances.

The challenge facing the analyst arises when the numbers of juveniles and of adults ringed are not known separately; instead, they are combined into one single total number. Yet the survival probability of the juvenile birds will be not only lower than their older counterparts, but (at least potentially) more akin to those of birds ringed prior to fledging.

### 2.3 Standard model

The standard model refers to the ring‐recovery model that estimates the survival and reporting probability of birds that were ringed early in their first year of life. Basic forms were proposed by Brownie et al. (1985) and Freeman and Morgan (1992) and have been used and extended in many studies since, see, for example, Thomson, Baillie, and Peach (1999), Gauthier and Lebreton (2008) and McCrea, Morgan, and Cole (2013).

The probability that a bird ringed in its first year of life in year *i* is recovered dead in year *t* is denoted by *P*_1,*i*,*t*_ with

$$P_{1,i,t}=\left\{\begin{array}{cc} \left(1-\phi_{1,t}\right)\lambda_{1,t}\; & \text{if}\;t=i\\ \phi_{1,i}\left(\prod_{k=i+1}^{t-1}\phi_{a,k}\right)\left(1-\phi_{a,t}\right)\lambda_{a,t} & \text{if}\;t>i \end{array}\right.$$

for *i* = 1, …, *n*_1_, *t* = *i*, …, *n*_2_.

Parameters can be estimated using maximum likelihood, and the likelihood function for the standard model for birds ringed in their first year of life is

(1) $$L_{S}\propto \left\{\prod_{i=1}^{n_{1}}\prod_{t=i}^{n_{2}}P_{1,i,t}^{N_{1,i,t}}\right\}\left\{\prod_{i=1}^{n_{1}}{\left(1-\sum_{t=i}^{n_{2}}P_{1,i,t}\right)}^{T_{1,i}-\sum_{t=i}^{n_{2}}N_{1,i,t}}\right\}.$$

Gauthier and Lebreton (2008) demonstrate how a ring‐recovery model can be written as a multi‐state model so that the program M‐Surge (Choquet, Reboulet, Pradel, Gimenez, & Lebreton, 2004) could be used to fit this model.

The model of Equation 1 can be used either for birds ringed as pulli or for birds ringed as pulli and juveniles; that is, birds ringed in their first year of life for which we know the ringing totals. While in practice it is not uncommon for data to be available for pulli alone, and thus modeled using Equation 1, they are unlikely to ever be available for fledged juveniles alone. The latter data will almost always be analyzed in conjunction with those of other age‐classes, as outlined in the following section.

### 2.4 Standard combined model

As stated by Robinson (2010), it is possible to fit a ring‐recovery model to the fledged birds’ data, with age‐specific ringing totals, when the total numbers of birds ringed as juveniles and as adults are known. The standard combined model can be used when there are separate data available on the total numbers of birds ringed in two different age classes. These two age classes are as follows: (a) birds in their first year of life (juveniles and/or pulli) and (b) adult birds. We use the word combined, as two different data sets are pooled together, and the likelihood function is obtained, under the assumption of independence, by multiplying two likelihood functions: the one for birds being ringed in their first year of life and the other for birds ringed as adults.

The probability that a bird ringed in the adult age class in year *i* is recovered dead in year *t* is denoted by *P*_*a*,*i*,*t*_ with

$$P_{a,i,t}=\left(\prod_{k=i}^{t-1}\phi_{a,k}\right)\left(1-\phi_{a,t}\right)\lambda_{a,t}$$

for *i* = 1, …, *n*_1_, *t* = *i*, …, *n*_2_.

If the fledged bird data are fully computerized, we have information on the number of birds ringed as juveniles, *T*_1,*i*_ and as adults, *T*_*a*,*i*_. Then, the likelihood function for the standard combined model is a straightforward product of that in Equation 1 and that for birds ringed as adults, that is,

(2) $$\begin{array}{cc} L_{\mathrm{SC}}\propto & \left\{\prod_{i=1}^{n_{1}}\prod_{t=i}^{n_{2}}P_{1,i,t}^{N_{1,i,t}}P_{a,i,t}^{N_{a,i,t}}\right\}\times \left\{\prod_{i=1}^{n_{1}}{\left(1-\sum_{t=i}^{n_{2}}P_{1,i,t}\right)}^{T_{1,i}-\sum_{t=i}^{n_{2}}N_{1,i,t}}\right.\\ & \left.\times {\left(1-\sum_{t=i}^{n_{2}}P_{a,i,t}\right)}^{T_{a,i}-\sum_{t=i}^{n_{2}}N_{a,i,t}}\right\}, \end{array}$$

See for example Brownie et al. (1985), and Freeman and Morgan (1992). We use the age category (1) for the *T*_*j*,*i*_ = *T*_1,*i*_ birds ringed as known juveniles that are by definition in their first year of life. If additional data on ringed pulli were available, this model can be used to fit these two data sets. If we were to add the pulli data, the age category (1) would include now birds ringed as juveniles (*j*), and as pulli (*p*). This is always assuming that the total number of birds ringed in each age category is known, and also that the same `first‐year’ survival probability applies to birds ringed as pulli or slightly older fledged juveniles. For a model separately estimating survival probabilities for the period immediately following fledging, see Thomson et al. (1999).

### 2.5 Historical data model

For records that are not computerized, only the total numbers of fledged birds ringed are known rather than the separate total numbers for juveniles and adults. The models used here for the historical fledged bird data are similar to the standard combined model for ring‐recovery data, but with the addition of a parameter that represents the unknown yearly proportion of birds ringed as juveniles. In these models, the proportion of juveniles ringed can be estimated as a constant parameter for all the years of study, or as a time‐dependent parameter allowing for variation between years. Robinson (2010) fixes this proportion to the mean observed in recent years, when the age at ringing data are recorded, and then tests sensitivity to this assumption. Here, we consider estimating this proportion. As this model is used to analyze historical data, in this paper it is termed the historical data model.

In the historical data model, the total numbers of birds ringed as juveniles, *T*_*j*,*i*_, and as adults, *T*_*a*,*i*_, are unknown. We only know the sum of both, *T*_*f*,*i*_ = *T*_*j*,*i*_ + *T*_*a*,*i*_, where *T*_*f*,*i*_ is the total number of fledged birds ringed in each year *i*, for *i* = 1, …, *n*_1_.

The historical data model has an additional parameter to be estimated.

- *π*_*t*_ is the proportion of fledged birds ringed as juveniles in year *t*, and (1 − *π*_*t*_) is the proportion of fledged birds ringed as adults.

The probability that a bird ringed in year *i* was a juvenile and was found dead in year *t* is

$$Q_{j,i,t}=\left\{\begin{array}{cc} \pi_{t}\left(1-\phi_{1,t}\right)\lambda_{1,t} & \text{if}\;t=i\\ \pi_{i}\phi_{1,i}\left(\prod_{k=i+1}^{t-1}\phi_{a,k}\right)\left(1-\phi_{a,t}\right)\lambda_{a,t} & \text{if}\;t>i \end{array}\right.$$

for *i* = 1, …, *n*_1_, *t* = *i*, …, *n*_2_. The probability that a bird ringed in year *i* was an adult and was recovered dead in year *t* is

$$Q_{a,i,t}=\left(1-\pi_{i}\right)\left(\prod_{k=i}^{t-1}\phi_{a,k}\right)\left(1-\phi_{a,t}\right)\lambda_{a,t}$$

for *i* = 1, …, *n*_1_, *t* = *i*, …, *n*_2_. The likelihood function for the historical data model for fledged birds is

(3) $$L_{H}=\left\{\prod_{i=1}^{n_{1}}\prod_{t=i}^{n_{2}}Q_{j,i,t}^{N_{j,i,t}}Q_{a,i,t}^{N_{a,i,t}}\right\}\times \left[\prod_{i=1}^{n_{1}}{\left\{1-\sum_{t=i}^{n_{2}}\left(Q_{j,i,t}+Q_{a,i,t}\right)\right\}}^{T_{f,i}-\sum_{t=i}^{n_{2}}\left(N_{j,i,t}+N_{a,i,t}\right)}\right].$$

There has been extensive work on mixture models dealing with unknown ages for capture–recapture data (see, e.g., Pledger, Efford, Pollock, Collazo, & Lyons, 2009; Pradel, 2009). Pledger and Schwarz (2002) developed mixture models in band‐recovery models (which is a reparameterization of the ring‐recovery model), and McCrea et al. (2013) examine age‐dependent mixture ring‐recovery models. Both mixture models assume that the group an individual belongs to, in this case juveniles and adults, is unknown for all individuals. In this paper however, we know which group some individuals belong to (the birds that were marked and recovered dead), but this information is unknown for the birds that were never recovered. Alternatively, this model could be written in a multi‐event format (Pradel, 2005), as we demonstrate in Supporting Information Appendix S3.

### 2.6 Historical combined data model

If there are separate data for birds ringed as pulli, the numbers of these will generally be known and, as in Robinson (2010), it is possible to use a standard model for the pulli data and a historical data model for the fledged birds of unknown age, in one combined analysis. This model will be referred to as the historical combined data model. Let *N*_*p*,*i*,*t*_ denote the number of pulli ringed in year *i* that were recovered dead in year *t*, and let *T*_*p*,*i*_ denote the total number of pulli ringed in year *i*. The probability that a pullus ringed in year *i* is recovered in year *t* is *P*_*p*,*i*,*t*_ = *P*_1,*i*,*t*_. The likelihood function for the historical combined data model is then

(4) $$\begin{array}{cc} L_{\mathrm{HC}}\propto & \left\{\prod_{i=1}^{n_{1}}\prod_{t=i}^{n_{2}}P_{p,i,t}^{N_{p,i,t}}Q_{j,i,t}^{N_{j,i,t}}Q_{a,i,t}^{N_{a,i,t}}\right\}\times \left[\prod_{i=1}^{n_{1}}{\left(1-\sum_{t=i}^{n_{2}}P_{p,i,t}\right)}^{T_{p,i}-\sum_{t=i}^{n_{2}}N_{p,i,t}}\right.\\ & \left.\times {\left\{1-\sum_{t=i}^{n_{2}}\left(Q_{j,i,t}+Q_{a,i,t}\right)\right\}}^{T_{f,i}-\sum_{t=i}^{n_{2}}\left(N_{j,i,t}+N_{a,i,t}\right)}\right]. \end{array}$$

### 2.7 Conditional model

In the case of unknown ringing totals, the conditional model, which conditions on the numbers of recovered individuals only, can be considered as an alternative to the historical data model. The conditional probabilities for birds ringed in year *i* that are recovered in year *t* in both age classes are

$$P_{j,i,t}^{C}=\frac{P_{j,i,t}}{\sum_{t=i}^{n_{2}}P_{j,i,t}}\;\text{and}\;P_{a,i,t}^{C}=\frac{P_{a,i,t}}{\sum_{t=i}^{n_{2}}P_{a,i,t}}$$

(McCrea et al., 2012). The likelihood function for the conditional model for the fledged birds is

(5) $$L_{C}=\prod_{i=1}^{n_{1}}\prod_{t=i}^{n_{2}}{\left(P_{j,i,t}^{C}\right)}^{N_{j,i,t}}{\left(P_{a,i,t}^{C}\right)}^{N_{a,i,t}}.$$

The conditional model is known to be parameter redundant, except when *λ* is constant, see Cole, Morgan, Catchpole, and Hubbard (2012).

### 2.8 Conditional combined model

As with the historical combined data model, separate data on pulli can be combined with the data on fledged birds. The standard model can be used for the pulli, and the conditional model can be used for fledged birds. This model, which we refer to as the conditional combined model, has likelihood

(6) $$L_{\mathrm{CC}}=\prod_{i=1}^{n_{1}}\prod_{t=i}^{n_{2}}{\left(P_{j,i,t}^{C}\right)}^{N_{j,i,t}}{\left(P_{a,i,t}^{C}\right)}^{N_{a,i,t}}P_{p,i,t}^{N_{p,i,t}}\times \prod_{i=1}^{n_{1}}{\left(1-\sum_{t=i}^{n_{2}}P_{p,i,t}\right)}^{T_{p,i}-\sum_{t=i}^{n_{2}}N_{p,i,t}}.$$

### 2.9 Model fitting

R code for fitting the historical data model is provided in Supporting Information Appendix S7. The historical data model could alternatively be fitted in the program E‐Surge (Choquet, Rouan, & Pradel, 2009), as explained in Supporting Information Appendix S3.

The models examined in this paper are summarized in Table 1. This can be interpreted as follows: The standard model with likelihood Equation 1 is implemented when the ringing total numbers available are for birds in their first year of life. The standard combined model, with likelihood Equation 2, is presented in two lines as this model can be used when we have two known separate ringing totals for juveniles and adults, or three known separate ringing totals for pulli, juveniles, and adults. The historical data model, with likelihood Equation 3, is implemented when the ringing totals available are for fledged birds; thus, we do not know the ringing totals for each age class separately. That is, we do not know how many birds were ringed as juveniles and how many birds were ringed as adults; instead, we know the sum of both numbers. The historical combined data model, with likelihood Equation 4, results as the combination of the standard and the historical data model. We use this model when the total number of birds ringed as pulli is known, but the total numbers of birds ringed as juveniles and as adults are unknown separately, and instead, we only have information on the sum of both numbers. The conditional and the conditional combined models with likelihood Equations 5 and 6 are the alternative models to the historical and historical combined data models.

**Table 1:** Description of the models defined above and used throughout the paper. The models in bold are the models developed in this paper and have an extra parameter *π*, the yearly proportion of birds ringed as fledged birds that are juveniles. Likelihood refers to the equation number of the likelihood function given in the paper. The last column specifies whether the total numbers of birds ringed per year per age category is known

Model name	Likelihood	Data on birds ringed as	Totals known?
Standard	(1)	First year of life	Yes
Standard combined	(2)	Fledged (juveniles and adults)	Yes
Standard combined	(2)	Pulli and fledged	Yes
**Historical**	(3)	Fledged	No
**Historical combined**	(4)	All age categories: Pulli and fledged	Pulli only
Conditional	(5)	Fledged	No
Conditional combined	(6)	All age categories: Pulli and fledged	Pulli only

### 2.10 Simulation study

To compare how the historical data model performs in practice, we provide two different simulation studies, which represent data on fledged birds. For all the simulations, all the parameters are kept constant over time. The first simulation study is for a population in which the adult survival is set to be homogeneous amongst the individuals. For the second simulation study, we consider a population with heterogeneous adult survival. We simulate two types of heterogeneous populations: an heterogeneous population in which adult survival *ϕ*_*a*,*i*_ varies individually, where *i* denotes a logit‐normal individual random effect; and an heterogeneous population formed by two subpopulations with two different adult survival probabilities: *ϕ*_*a*,*A*_ and *ϕ*_*a*,*B*_. The simulation studies for the heterogeneous populations allow us to test how the proposed models perform in practice when there is individual variation in survival caused by factors other than age.

For the homogeneous population, in each simulation 100 data sets are simulated from the standard combined model with 1,000 birds ringed with a constant proportion *π* of the birds ringed in their first year of life, and (1 − *π*) of the birds ringed as adults. In this model, there are separate constant survival probabilities for first‐year and adult birds, *ϕ*_1_ and *ϕ*_*a*_, respectively, and a constant reporting probability, *λ*. Specific details for the simulation studies for the heterogeneous populations can be found in Supporting Information Appendix S5.

Both the standard combined model and the historical data model described above are then fitted to each simulated data set. In the standard combined model, the total numbers of birds ringed in each of the two age classes are known, whereas in the historical model, only the total number of birds ringed is used. Otherwise, the forms of the two models are identical and match the form used in generating the data. We also fit the historical data model with *π* fixed either to the true value or to an arbitrary wrong value.

The simulation results for 10 years of study for an homogeneous population are given in Table 2, and further results for five and 20 years of study can be found in Supporting Information Appendix S4 (Tables S7 and S8). The heterogeneous population results for five, 10, and 20 years of study are given in Supporting Information Appendix S5 (Tables S9–S23). By simulating data for different study lengths, we show how the magnitude of parameter bias is affected by the length of the study. We compare model performance by looking at the bias, the standard deviation, and the mean squared error of the parameters across 100 simulations.

**Table 2:** Simulation study for 10 years of ring‐recovery data. The first column specifies the type of model, with Stand. Comb. short for standard combined and Hist. short for historical. In this first column, the last two rows contain information for the models in which the proportion parameter was fixed and the values used. The remaining columns contain the average parameter estimate (par est) and the average standard error, given in parentheses, along with the mean squared error (MSE)

	*ϕ*_1_		*ϕ*_*a*_		*λ*		*π*	
	par est	MSE	par est	MSE	par est	MSE	par est	MSE
True value	0.50	–	0.60	–	0.05	–	0.40	–
Stand. Comb.	0.50 (0.04)	0.0014	0.60 (0.02)	0.0005	0.05 (0.002)	0.0000	–	–
Hist.	0.50 (0.04)	0.0014	0.60 (0.02)	0.0005	0.05 (0.002)	0.0000	0.40 (0.02)	0.0006
Hist. *π* = 0.40	0.50 (0.04)	0.0014	0.60 (0.02)	0.0005	0.05 (0.002)	0.0000	–	–
Hist. *π* = 0.20	0.46 (0.04)	0.0024	0.63 (0.03)	0.0010	0.05 (0.001)	0.0000	–	–

The simulation studies show that for an homogeneous population, the historical data model gives almost identical parameter estimates for survival and recovery to the standard combined model. The same is true for the heterogeneous populations. Both homogeneous and heterogeneous populations result in unbiased estimates of the proportion of birds ringed in their first year of life, *π*. This demonstrates that the historical data model can be used to estimate the additional parameter *π* as well as the survival and recovery parameters as accurately as the standard ring‐recovery model, which requires additional information.

If *π* is fixed at the true value, then the historical data model with a fixed *π* also performs just as well, as would be expected. However, if *π* is not fixed at the true value then there is bias in the estimation of the survival parameters, the mean squared error is bigger, and the standard error is higher than in other models. For example, when *π* is fixed at a lower value than the true value, fewer birds are estimated to survive their first year, and to retain a match to the subsequent numbers recovered adult survival is increased in compensation. For constant *π*, the bias decreases as the number of years of ringing and recovery increases. It is therefore recommended that the historical data model is used rather than fixing *π*.

Further simulation studies for different parameter estimates show very similar results and the recommendation from these simulation studies, and Table 3 is that if total ringing numbers in each category are unavailable, it is preferable to use the historical data model and estimate the proportion in each age class.

**Table 3:** Parameter redundancy results. The main body of the table gives the deficiency. The first row specifies the type of model, with Stand. short for standard, Cond. short for conditional, and Cond. Comb. short for conditional combined. The combined column is applicable for both the combined historical data model and the combined standard model. The second row specifies which types of data the model is suitable for, either pulli, fledged (Fl), or both combined (Pulli + Fl). The third row specifies the proportion parameter, which is either constant (*π*) or time‐dependent (*π*_*t*_), or not included in that type of model (–). The first column specifies the parameters in the model. Results here are for the same number of years of ringing as recovery, *n* = *n*_1_ = *n*_2_. The results for the standard model, that can be used for the pulli data alone, come from Cole et al. (2012)

Model	Stand.	Historical		Combined	Cond.	Cond. Comb.
Data set(s)	Pulli	Fl		Pulli + Fl	Fl	Pulli + Fl
Proportion	–	*π*	*π*_*t*_	*π* or *π*_*t*_	–	–
*ϕ*_1_, *ϕ*_*a*_, *λ*	0	0	0	0	0	0
*ϕ*_1_, *ϕ*_*a*_, *λ*_*t*_	0	0	0	0	2	0
*ϕ*_1_, *ϕ*_*a*_, *λ*_1_, *λ*_*a*_	1	1	0	0	2	1
*ϕ*_1_, *ϕ*_*a*_, *λ*_1,*t*_, *λ*_*a*,*t*_	1	1	0	0	3	1
*ϕ*_1_, *ϕ*_*a*,*t*_, *λ*	0	0	0	0	0	0
*ϕ*_1_, *ϕ*_*a*,*t*_, *λ*_*t*_	0	0	0	0	3	0
*ϕ*_1_, *ϕ*_*a*,*t*_, *λ*_1_, *λ*_*a*_	1	1	0	0	2	1
*ϕ*_1_, *ϕ*_*a*,*t*_, *λ*_1,*t*_, *λ*_*a*,*t*_	2	2	3	1	*n* + 2	3
*ϕ*_1,*t*_, *ϕ*_*a*_, *λ*	0	0	0	0	0	0
*ϕ*_1,*t*_, *ϕ*_*a*_, *λ*_*t*_	0	0	0	0	2	0
*ϕ*_1,*t*_, *ϕ*_*a*_, *λ*_1_, *λ*_*a*_	0	0	0	0	2	0
*ϕ*_1,*t*_, *ϕ*_*a*_, *λ*_1,*t*_, *λ*_*a*,*t*_	2	2	3	1	*n* + 1	3
*ϕ*_1,*t*_, *ϕ*_*a*,*t*_, *λ*	0	0	0	0	1	0
*ϕ*_1,*t*_, *ϕ*_*a*,*t*_, *λ*_*t*_	2	1	2*n* + 1	1	*n* + 1	2
*ϕ*_1,*t*_, *ϕ*_*a*,*t*_, *λ*_1_, *λ*_*a*_	0	0	0	0	3	0
*ϕ*_1,*t*_, *ϕ*_*a*,*t*_, *λ*_1,*t*_, *λ*_*a*,*t*_	*n* + 1	3	*n* + 2	2	2*n*	*n* + 2

### 2.11 Parameter redundancy

In ring‐recovery models, it is common to have two or more parameters in a model that only appear in the likelihood as a product or in a similar function of the parameters, and which hence cannot be estimated independently (see, e.g., Chapter 10, McCrea & Morgan, 2015). This phenomenon is known as parameter redundancy or the model can be described as nonidentifiable. A parameter redundant model can be reparameterized into one with a smaller number of parameters, and it is not possible to estimate all the parameters in the full model, without first reparameterizing, or imposing some constraint on the model.

We used parameter identifiability theory to determine which parameters in the historical and combined models can be estimated, and these are compared to the parameters that can be estimated in the standard ring‐recovery model, which are given by Cole et al. (2012). The theory underlying parameter identifiability is now extensive and complex; we shall provide only a brief background here and refer the reader to published material (Cole, Morgan, & Titterington, 2010). Such theory has been used to determine the utility of a range of models in ecology (Allen, Satterthwaite, Hankin, Cole, & Mohr, 2017; Cole, 2012; Cole & Morgan, 2010; Cole et al., 2012; Hubbard, Cole, & Morgan, 2014). Ecological models that have been examined include ring‐recovery models (Catchpole & Morgan, 1997; Cole et al., 2010, 2012), capture–recapture models (Catchpole & Morgan, 1997; Catchpole, Morgan, & Freeman, 1998; Cole et al., 2010; Gimenez, Viallefont, Catchpole, Choquet, & Morgan, 2004; Hubbard et al., 2014), capture–recapture–recovery models (Hubbard et al., 2014), multi‐state (Cole, 2012; Gimenez, Choquet, & Lebreton, 2003) and state‐space models (Cole & McCrea, 2016). In short, symbolic algebra is used to determine the rank of a matrix derived from the model. This rank is the number of parameters that can be estimated in the model. If all the parameters in a model can be estimated, then this rank will be the same as the number of parameters. Otherwise, when the rank is less than the number of parameters a model is described as parameter redundant, and not all of the parameters can be estimated. Here, we present the deficiency of the model, which is the difference between the number of parameters and the rank. A deficiency of zero indicates the model is not parameter redundant, and in theory, all the parameters can be estimated. A deficiency of more than zero indicates the model is parameter redundant and cannot be fitted in that form. In a parameter redundant model, it may still be possible to estimate some of the parameters, which can be found using further matrix algebra, along with a reparameterization of the model that can be estimated (Catchpole et al., 1998; Cole et al., 2010). This method is most practically executed in a matrix algebra software such as Maple (Catchpole, Morgan, & Viallefont, 2002; Cole et al., 2010). Alternatively, a hybrid symbolic–numeric method can be used (Choquet & Cole, 2012), where the rank is found numerically.

Parameter redundancy can also be caused by the data. In ring‐recovery models, this can occur when there are several N_*,*i*,*t*_ equal to zero. Cole et al. (2012) show that one N_*,*i*,*t*_ for *t* = *i*, …, *n*_2_ can be zero for each *t*, and parameter redundancy results will always remain unchanged. Other patterns of zeroes can, but do not always, result in parameter redundancy, as demonstrated in Cole et al. (2012). Cole et al. (2012) used the symbolic method to check parameter redundancy cause by the data, but it could also be checked using the hybrid symbolic–numeric method (Choquet & Cole, 2012), which is available in E‐Surge (Choquet et al., 2009). Therefore, if the multi‐event format of Supporting Information Appendix S3 is used it is possible to check for parameter redundancy caused by the data automatically.

Models can also behave as if they were parameter redundant for certain data sets. This is known as near‐redundancy and normally occurs if there is a nested parameter redundant model, and the maximum likelihood parameter estimates are close to those of the nested model (Catchpole, Kgosi, & Morgan, 2001). Catchpole et al. (2001) show how near‐redundancy can be detected by examining the smallest eigenvalue of the Hessian matrix. A value that is close to zero indicates near‐redundancy. The sparse Sandwich Tern data set is used to explore the potential effects of near‐redundancy on the models presented here.

## 3 RESULTS

### 3.1 Parameter redundancy

All of the models that have a parameter deficiency of zero for the standard model also have a deficiency of zero for the historical data model (Table 3). When the pulli data are combined with the fledged birds, a combined model results in more models having a deficiency of zero. The combined historical data model is shown to have identical deficiency to the standard combined model, so in terms of parameter redundancy, it does not matter whether or not the data have been fully computerized.

The alternative to the historical data model is the conditional model. However, for ring‐recovery data most conditional models are parameter redundant. When the pulli data are combined with the fledged bird data, the conditional combined model does not do any better than the pulli data alone, in terms of parameter redundancy. There is only a deficiency of zero if the pulli model also has a deficiency of zero. Therefore, in terms of parameter redundancy there is no gain from using the conditional model. In terms of parameter redundancy, the best model to use for historical data is the historical data model.

Being able to show theoretically that a model is identifiable, however, is no guarantee that for some specific data the model would not be parameter or near parameter redundant. We also consider parameter redundancy in practice by using simulation and considering data on two different ring‐recovery data sets. Below, we run a simulation study to show that the historical data model gives almost identical parameter estimates as the standard model. We explore the Blackbird data set, where we know the total number of birds ringed by age‐class, and compare the performance of the historical data model with the standard model. Then, similar to Robinson (2010) we use a subset of Sandwich Tern data, where we do not know these totals.

### 3.2 Blackbird case study

Using the Blackbird data set for comparison purposes, we have run two different analyses. In the first analysis, we sum together the total of birds ringed in the fledged category (and we assume that the numbers of juveniles and adults ringed each year were unknown, and only the sum of both was available), then we fit historical data models to this data set. In the second analysis, we use all the available data (using either two or three, known ringing totals, i.e., juveniles and adults, or pulli, juveniles, and adults), and we fit standard combined models. The parameter estimates for survival and reporting probabilities for the historical and historical combined data model, and their standard errors, are almost identical to those for the standard combined models (Table 4). However, the first‐year survival probability, *ϕ*_1_, is slightly smaller for the models that analyze pulli data. Birds ringed as pulli are younger than those fledged juveniles; thus, their survival is likely to be lower. Therefore, adding the pulli data to the first‐year age class brings the survival probability *ϕ*_1_ down.

**Table 4:** Estimates of survival and reporting probabilities for Blackbird data using the standard combined, historical, and historical combined data model. The parameter estimates are given alongside standard errors in parentheses. The first row specifies the type of model, with Stand. Comb. and Hist. Comb. short for standard and historical combined. The second row specifies the data used for the analysis, with Juv, Ad, and Fl short for ringing totals for juvenile, adult, and fledged birds

Model	Stand. Comb.	Historical	Stand. Comb.	Hist. Comb.
Data set(s)	Juv, Ad	Fl	Pulli, Juv + Ad	Pulli, Fl
*ϕ*_1_	0.5925 (0.0085)	0.5933 (0.0085)	0.5451 (0.0067)	0.5454 (0.0067)
*ϕ*_*a*_	0.6965 (0.0050)	0.6958 (0.0049)	0.6915 (0.0045)	0.6909 (0.0044)
*λ*	0.0375 (0.0005)	0.0375 (0.0005)	0.0361 (0.0004)	0.0361 (0.0004)
*π*		0.5760 (0.0066)		0.5741 (0.0066)

**Figure 1:**
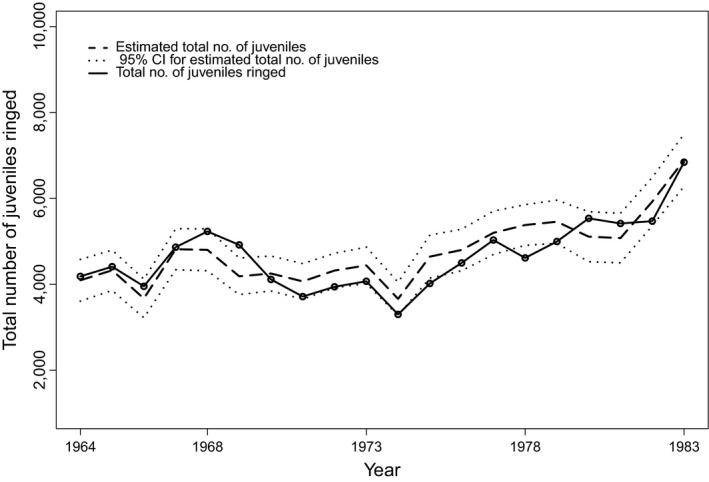
Comparison between the total number of juvenile Blackbirds ringed per year, indicated by the continuous line, and the estimated total number of juveniles, indicated by the dashed line, obtained from the time‐dependent proportion of juveniles (*π*_*t*_), for the historical combined data model with parameters (*ϕ*_1_, *ϕ*_*a*_, *λ*_*t*_, *π*_*t*_). The dotted lines represent the 95% confidence interval

Tables 5 and 6 compare the best models in terms of the Akaike information criterion (AIC; Akaike, 1974) for the standard combined and the historical data models. To compare the model selection for the historical and the standard combined models, we check if the dependencies for the survival and recovery parameters for the best models agree. For example, the standard model with parameters (*ϕ*_1_, *ϕ*_*a*_, *λ*_*t*_) would be equivalent, demographically, to the historical data models with parameters (*ϕ*_1_, *ϕ*_*a*_, *λ*_*t*_, *π*), and (*ϕ*_1_, *ϕ*_*a*_, *λ*_*t*_, *π*_*t*_). Table 5 shows that the best models for the historical and the standard combined agree; the same parameterization is chosen for the two different models. Moreover, Table 6 shows that although the same best (demographic) models are chosen for the historical combined and the standard combined models, the order of preference differs. The best model for the historical combined data model has parameters (*ϕ*_1_, *ϕ*_*a*_, *λ*_1,*t*_, *λ*_*a*,*t*_, *π*), followed very closely by the models with parameters the (*ϕ*_1,*t*_, *ϕ*_*a*_, *λ*_*t*_, *π*) and (*ϕ*_1_, *ϕ*_*a*_, *λ*_*t*_, *π*_*t*_); for the standard combined model, the best model has parameters (*ϕ*_1_, *ϕ*_*a*_, *λ*_*t*_), followed, some distance behind, by the model with parameters (*ϕ*_1_, *ϕ*_*a*_, *λ*_1,*t*_, *λ*_*a*,*t*_). Nonetheless, the difference in AIC between the top historical data models is very small and seems to show that the age dependency in the recovery probability does not contribute significantly to the model when the proportion of juvenile birds ringed is not known, but estimated. Finally, Figure 1 shows that the total number of juvenile Blackbirds estimated from the fledged bird data set for the best historical combined data model with parameters (*ϕ*_1_, *ϕ*_*a*_, *λ*_*t*_, *π*_*t*_) is very similar to the real number of ringed juvenile Blackbirds.

**Table 5:** Comparison between the historical ring‐recovery and standard combined model selection for Blackbirds. The first and the third columns show the models fitted and the parameterization used

Historical		Standard combined	
Fl	*Δ* AIC	Juveniles + adults	*Δ* AIC
*ϕ*_1_, *ϕ*_*a*_, *λ*_*t*_, *π*_*t*_	0.00	*ϕ*_1_, *ϕ*_*a*_, *λ*_*t*_	0.00
*ϕ*_1_, *ϕ*_*a*,*t*_, *λ*_*t*_, *π*_*t*_	3.68	*ϕ*_1_, *ϕ*_*a*,*t*_, *λ*_*t*_	11.89
*ϕ*_1_, *ϕ*_*a*_, *λ*_*t*_, *π*	6.65		
*ϕ*_1_, *ϕ*_*a*,*t*_, *λ*_*t*_, *π*	18.30		
*ϕ*_1,*t*_, *ϕ*_*a*_, *λ*_*t*_, *π*	20.30	*ϕ*_1,*t*_, *ϕ*_*a*_, *λ*_*t*_	23.87
*ϕ*_1,*t*_, *ϕ*_*a*_, *λ*_*t*_, *π*_*t*_	22.59		
*ϕ*_1_, *ϕ*_*a*_, *λ*,* π*	148.56	*ϕ*_1_, *ϕ*_*a*_, *λ*	140.58

**Table 6:** Comparison between historical combined and standard combined ring‐recovery model selection for Blackbirds. The first and the third columns show the models fitted and the data used, with Juv, Ad, and Fl short for ringing totals for juvenile, adult, and fledged birds

Historical Combined Pulli + Fl	*Δ* AIC	Standard combined Pulli + Juv + Ad	*Δ* AIC
*ϕ*_1_, *ϕ*_*a*_, *λ*_1,*t*_, *λ*_*a*,*t*_, *π*	0.00	*ϕ*_1_, *ϕ*_*a*_, *λ*_1,*t*_, *λ*_*a*,*t*_	26.48
*ϕ*_1,*t*_, *ϕ*_*a*_, *λ*_*t*_, *π*	2.30	*ϕ*_1,*t*_, *ϕ*_*a*_, *λ*_*t*_	38.38
*ϕ*_1_, *ϕ*_*a*_, *λ*_*t*_, *π*_*t*_	2.60	*ϕ*_1_, *ϕ*_*a*_, *λ*_*t*_	0.00
*ϕ*_1_, *ϕ*_*a*_, *λ*_1,*t*_, *λ*_*a*,*t*_, *π*_*t*_	5.10		
*ϕ*_1_, *ϕ*_*a*_, *λ*_*t*_, *π*	5.80		
*ϕ*_1_, *ϕ*_*a*,*t*_, *λ*_*t*_, *π*_*t*_	17.70	*ϕ*_1_, *ϕ*_*a*,*t*_, *λ*_*t*_	30.40
*ϕ*_1_, *ϕ*_*a*_, *λ*,* π*	146.10	*ϕ*_1_, *ϕ*_*a*_, *λ*	148.33

### 3.3 Sandwich tern case study

For longer‐lived species, juvenile survival may be substantially lower, so we analyze Sandwich Tern data for which the separate ringing totals are unknown for juveniles and adults. This is a longer‐lived species than the Blackbird, but it is rarer and the data are consequently sparser (Supporting Information Appendix S2). When fitted, most of the historical data models prove to be parameter redundant or near‐redundant. In fact, the only model that did not present any identifiability issues was that in which all the parameters were kept constant. However, when looking at the parameter estimates, the first‐year survival probability was estimated much higher than the adult survival probability, which does not appear realistic. Moreover, for most of the models we were unable to maximize the likelihood function.

The results described above are a clear example of parameter redundancy caused by the data. The issue of parameter redundancy improved when adding the pulli data to the historical data model by fitting the historical combined data model. As a result, many more models could be fitted; however, there were only three models that did not present any sign of parameter redundancy. The parameter estimates for these models can be found in Table 7 and in Supporting Information Appendix S6 (Figures S1 and S2). Furthermore, when adding the pulli data, the constant model presented more reliable results, with a higher survival probability for adults than for first years, and with parameters estimates closer to those expected based on the results obtained when fitting the standard model to pulli data alone.

**Table 7:** Historical combined ring‐recovery model selection and parameter estimates for Sandwich Terns for 1970–1990. The standard errors are given in parentheses. The parameter estimates for the time‐dependent parameters can be found in Supporting Information Appendix S6

Parameters	*Δ* AIC	*ϕ*_1_	*ϕ*_*a*_	*λ*	*π*
*ϕ*_1_, *ϕ*_*a*_, *λ*_*t*_, *π*	0.00	0.74 (0.016)	0.87 (0.010)	–	0.32 (0.076)
*ϕ*_1_, *ϕ*_*a*_, *λ*,* π*	27.67	0.73 (0.015)	0.87 (0.010)	0.02 (0.001)	0.32 (0.076)
*ϕ*_1,*t*_, *ϕ*_*a*_, *λ*,* π*	34.01	–	0.86 (0.010)	0.02 (0.001)	0.32 (0.076)

Sandwich Terns are bigger than Blackbirds, and most do not begin to breed until their third year of life; thus, a more complex age structure may well be more realistic, although these extra parameters may hinder further parameter identifiability. Robinson (2010) explores a model with three age classes, where he estimates survival and reporting probabilities for first‐year birds, birds in their second or third year, and older birds. We do not have data on ringing totals for birds ringed in their second or third year of life, though these are possibly few as the young birds relocate to West Africa before returning to breed. As for most species, we also do not know the age of the adult Sandwich Terns at ringing. If this information was known, for this or a similarly long‐lived species, a better alternative would be to model this age structure with the approach presented in this paper in combination with the age‐dependent mixture model proposed by McCrea et al. (2013).

## 4 DISCUSSION

We have presented a model that estimates age‐dependent survival probabilities from ring‐recovery data where the number of individuals ringed in each age class is unknown. Using identifiability theory, we have shown that it is possible to estimate the proportion of individuals in each age class. Using simulation and a data set where the ringing numbers are known by age category, we have demonstrated that the historical data model gives almost identical parameter estimates as the standard ring‐recovery model. Therefore, we have shown that it is possible to fit useful age‐dependent survival models to historical ringing data even though the data may not have been fully computerized.

This new model provides an extension to the analysis of UK ringing data of Robinson (2010), where the proportion in each age category was fixed. The new historical data model has the advantage that, by estimating rather than assuming age‐specific ratios at ringing, estimates of precision and hypothesis tests are more reliable—if a constraint is imposed unnecessarily, there is bias in estimating survival and the resulting standard errors may overestimate the uncertainty in selecting this value, as demonstrated in the simulation study.

Although these results were motivated by an analysis of UK ringing data (Robinson, 2010), many national ringing schemes, in Europe and North America (Tautin, 2008), face a similar challenge. For European schemes, details of birds ringed and subsequently recovered have been routinely collated and are accessible for analysis. The Euring Data Bank (EDB) currently holds in excess of 10 million such records (du Feu, Clark, Schaub, Fiedler, & Baillie, 2016). Moreover, although here we just look at estimating probabilities for two age categories, first‐year and adult birds, the historical data model can be extended with the addition of an age‐mixture model (McCrea et al., 2013) to incorporate other age dependencies that accommodate for differences in breeding age between species.

These results indicate that it is possible to incorporate age‐specific variation in models of survival, further unlocking the potential of a valuable historical data archive compiled over several decades to better characterize temporal dynamics in the population processes of many species. Such data are, of course, often the only source of demographic data ever likely to be available as a benchmark against which to compare estimates from more recent years and different climatic and agricultural contexts.

## CONFLICT OF INTEREST

None declared.

## Data Availability

The data files and the R code have been uploaded and will be available at the Kent Academic Repository (KAR) https://www.kent.ac.uk/library/research/kar/index.html.
