# Supplementary material for: Estimating age‐dependent survival from age‐aggregated ringing data—extending the use of historical records
Source: Ecol Evol. 2019 Feb 5;9(2):769–79. doi: 10.1002/ece3.4820 (PMC6362446; doi:10.1002/ece3.4820)
Supplement: Supplementary file 1 [file ECE3-9-769-s001.pdf]

Short Title - On age-aggregated ringing data

Supporting Information - Estimating age-dependent survival from age-aggregated ringing data - extending the use of historical records

Marina Jiménez-Muñoz[1*], Diana J. Cole[1], Stephen N. Freeman[2], Robert A. Robinson[3], Stephen R. Baillie[3], Eleni Matechou[1]

[1] School of Mathematics, Statistics and Actuarial Science, University of Kent, Canterbury, CT2 7FS, UK.

[2] Centre for Ecology & Hydrology, Wallingford, OX10 8BB, UK.

[3]British Trust for Ornithology, The Nunnery, Thetford, IP24 2PU, UK.

S1 - Blackbird Data

Ring-recovery m-arrays for Blackbird data taken from Robinson *et al.* (2012). Table S1 contains the ringing and recovery totals for the birds ringed as pulli. Table S2 contains the ringing and recovery totals for birds ringed as juveniles and adults respectively.

Table S1: Ring-recovery data for pulli Blackbirds for the years 1964-1983.

Year of	Year of recovery																				Total
ringing	1	2	3	4	5	6	7	8	9	10	11	12	13	14	15	16	17	18	19	20	ringed
**Ringed as pulli**																					
1964	52	15	10	2	8	5	3	1	0	1	0	0	0	0	0	0	0	0	0	0	2488
1965	0	74	30	14	18	7	12	2	1	3	1	1	0	0	0	0	0	0	0	0	3583
1966	0	0	78	29	20	11	4	4	2	0	2	1	0	1	0	0	0	0	0	0	4518
1967	0	0	0	67	22	12	4	7	4	2	2	1	4	1	2	0	0	0	0	0	4315
1968	0	0	0	0	101	30	10	8	8	6	3	6	1	1	1	0	0	0	0	0	4347
1969	0	0	0	0	0	81	27	10	11	10	4	1	3	1	3	1	0	0	1	0	4517
1970	0	0	0	0	0	0	58	15	8	5	4	4	3	2	0	0	1	1	0	0	3448
1971	0	0	0	0	0	0	0	55	19	11	10	6	6	5	3	0	1	1	0	0	3461
1972	0	0	0	0	0	0	0	0	67	21	14	6	7	6	1	0	2	2	0	1	3745
1973	0	0	0	0	0	0	0	0	0	54	26	6	8	2	4	2	1	0	1	1	3139
1974	0	0	0	0	0	0	0	0	0	0	42	17	5	9	3	1	1	3	0	0	2811
1975	0	0	0	0	0	0	0	0	0	0	0	57	12	8	4	2	5	0	0	1	3166
1976	0	0	0	0	0	0	0	0	0	0	0	0	58	15	10	5	8	2	3	2	3141
1977	0	0	0	0	0	0	0	0	0	0	0	0	0	64	13	7	7	4	3	1	3535
1978	0	0	0	0	0	0	0	0	0	0	0	0	0	0	60	21	8	10	9	1	3646
1979	0	0	0	0	0	0	0	0	0	0	0	0	0	0	0	62	15	11	4	9	3918
1980	0	0	0	0	0	0	0	0	0	0	0	0	0	0	0	0	54	15	8	9	3403
1981	0	0	0	0	0	0	0	0	0	0	0	0	0	0	0	0	0	57	18	8	3510
1982	0	0	0	0	0	0	0	0	0	0	0	0	0	0	0	0	0	0	41	14	2927
1983	0	0	0	0	0	0	0	0	0	0	0	0	0	0	0	0	0	0	0	70	4150

Table S2: Ring-recovery data for fledged Blackbirds for the years 1964-1983.

Year of ringing	Year of recovery 1	2	3	4	5	6	7	8	9	10	11	12	13	14	15	16	17	18	19	20	Total ringed
**Ringed as juveniles**																					
1964	114	39	17	12	10	12	8	5	3	3	0	0	0	0	0	0	0	0	0	0	4408
1965	0	93	26	31	19	16	6	7	3	6	1	1	0	0	0	1	0	0	0	0	4621
1966	0	0	70	32	19	20	8	5	4	4	2	1	1	1	0	0	0	0	0	0	4121
1967	0	0	0	85	34	21	25	13	11	4	2	4	3	0	2	0	0	0	0	0	5069
1968	0	0	0	0	75	30	34	18	16	8	11	5	6	2	1	0	0	0	0	0	5438
1969	0	0	0	0	0	76	30	28	25	20	9	7	5	5	2	2	0	0	0	0	5127
1970	0	0	0	0	0	0	61	27	23	21	13	6	7	4	3	2	0	0	0	0	4281
1971	0	0	0	0	0	0	0	53	34	15	14	11	10	4	8	1	1	0	0	0	3866
1972	0	0	0	0	0	0	0	0	74	21	17	14	11	8	9	6	3	1	1	0	4108
1973	0	0	0	0	0	0	0	0	0	81	28	25	19	14	13	9	6	5	2	2	4275
1974	0	0	0	0	0	0	0	0	0	0	67	16	11	11	11	10	2	1	0	0	3429
1975	0	0	0	0	0	0	0	0	0	0	0	71	33	17	15	7	6	1	5	0	4175
1976	0	0	0	0	0	0	0	0	0	0	0	0	68	28	17	11	13	5	8	4	4652
1977	0	0	0	0	0	0	0	0	0	0	0	0	0	82	34	23	13	9	7	3	5202
1978	0	0	0	0	0	0	0	0	0	0	0	0	0	0	74	30	13	15	8	7	4762
1979	0	0	0	0	0	0	0	0	0	0	0	0	0	0	0	75	29	17	17	13	5148
1980	0	0	0	0	0	0	0	0	0	0	0	0	0	0	0	0	61	35	24	13	5669
1981	0	0	0	0	0	0	0	0	0	0	0	0	0	0	0	0	0	62	31	22	5532
1982	0	0	0	0	0	0	0	0	0	0	0	0	0	0	0	0	0	0	59	36	5566
1983	0	0	0	0	0	0	0	0	0	0	0	0	0	0	0	0	0	0	0	99	6942
**Ringed as adults**																					
1964	39	18	19	8	9	3	0	2	5	0	0	0	0	0	0	0	0	0	0	0	1994
1965	0	44	23	16	15	16	4	6	1	1	0	0	1	0	0	0	0	0	0	0	2471
1966	0	0	32	27	10	11	10	6	3	4	1	1	0	0	0	0	0	0	0	0	2459
1967	0	0	0	42	29	24	12	10	6	4	4	4	3	0	0	0	0	0	0	0	3131
1968	0	0	0	0	38	31	15	14	11	4	6	5	1	0	0	0	0	0	0	0	2991
1969	0	0	0	0	0	62	29	18	20	15	12	8	4	2	2	0	0	0	0	0	3069
1970	0	0	0	0	0	0	47	25	21	20	18	7	3	3	0	2	0	0	0	0	3202
1971	0	0	0	0	0	0	0	32	39	20	15	11	13	3	2	4	0	0	0	0	3450
1972	0	0	0	0	0	0	0	0	38	28	25	20	14	9	3	3	0	0	1	0	3784
1973	0	0	0	0	0	0	0	0	0	42	33	24	17	10	7	5	1	1	0	0	3903
1974	0	0	0	0	0	0	0	0	0	0	35	28	26	16	11	3	6	2	0	0	3795
1975	0	0	0	0	0	0	0	0	0	0	0	37	25	15	10	9	10	4	4	0	5045
1976	0	0	0	0	0	0	0	0	0	0	0	0	36	31	19	12	8	6	1	2	4432
1977	0	0	0	0	0	0	0	0	0	0	0	0	0	41	23	17	15	11	2	2	4181
1978	0	0	0	0	0	0	0	0	0	0	0	0	0	0	38	16	18	13	10	8	4156
1979	0	0	0	0	0	0	0	0	0	0	0	0	0	0	0	41	31	28	7	9	4052
1980	0	0	0	0	0	0	0	0	0	0	0	0	0	0	0	0	50	38	23	16	4632
1981	0	0	0	0	0	0	0	0	0	0	0	0	0	0	0	0	0	48	26	30	4924
1982	0	0	0	0	0	0	0	0	0	0	0	0	0	0	0	0	0	0	25	21	4126
1983	0	0	0	0	0	0	0	0	0	0	0	0	0	0	0	0	0	0	0	41	4670

S2 - Sandwich Tern Data

Ring-recovery m-arrays for Sandwich Tern data. Table S3 contains the ringing and recovery totals for birds ringed as pulli. Table S4 contains the recovery totals for birds ringed as juveniles and adults respectively. The total number of birds ringed in each age category are not known, and are represented with a dash (-). Table S5 contains the total number of birds ringed as fledged birds (juveniles and adults).

Table S3: Ring-recovery data for pulli Sandwich Terns for the years 1970-1990.

Year of	Year of recovery																					Total
ringing	1	2	3	4	5	6	7	8	9	10	11	12	13	14	15	16	17	18	19	20	21	ringed
											**Ringed as pulli**											
1970	23	17	1	1	4	7	0	1	1	2	1	2	3	0	1	1	1	2	0	2	2	4021
1971		17	7	7	5	6	4	3	1	2	2	4	1	0	0	0	1	1	2	3	4	3780
1972			24	10	4	1	2	1	0	0	1	0	2	2	0	1	0	1	0	1	2	3583
1973				18	15	1	4	4	2	3	4	4	1	2	0	1	3	0	0	2	1	3903
1974					17	6	2	1	3	4	3	4	3	0	2	1	0	0	2	2	5	3561
1975						24	10	5	3	4	0	3	2	1	0	2	2	2	2	1	4	3532
1976							15	10	2	4	2	0	1	0	1	1	0	1	1	0	0	2378
1977								18	3	7	1	1	0	1	1	0	0	0	0	0	1	2286
1978									10	12	4	4	2	5	2	2	0	2	2	1	4	3239
1979										14	2	0	1	1	0	1	1	1	1	0	2	2429
1980											18	6	4	7	6	5	3	2	0	3	6	4550
1981												17	13	4	3	3	2	3	0	2	2	4083
1982													17	7	6	2	1	2	1	3	2	4464
1983														19	10	2	2	3	2	1	4	3723
1984															8	7	3	5	2	3	6	3378
1985																4	6	4	1	1	6	2373
1986																	6	7	3	3	6	3378
1987																		5	5	4	4	1799
1988																			7	4	3	1848
1989																				4	5	2542
1990																					12	2158

Table S4: Number of fledged Sandwich Terns recovered dead from 1970-1990. The number of birds ringed for each category is not known. The total amount of birds ringed for the fledged birds is given in Table S5.

Year of ringing	Year of recovery 1	2	3	4	5	6	7	8	9	10	11	12	13	14	15	16	17	18	19	20	21	Total ringed
**Ringed as juveniles**																						
1970	0	0	0	0	0	0	0	0	0	0	0	0	0	0	0	0	0	0	0	0	0	-
1971		0	0	0	0	0	1	0	0	0	0	0	0	0	0	0	0	0	0	0	0	-
1972			0	0	0	0	0	0	0	0	0	0	0	0	0	0	0	0	0	0	0	-
1973				0	0	0	0	0	0	0	0	0	0	0	0	0	0	0	0	0	0	-
1974					0	0	0	0	0	0	0	0	0	0	0	0	0	0	0	0	0	-
1975						0	0	0	0	0	0	0	0	0	0	0	0	0	0	0	0	-
1976							0	0	0	0	0	0	0	0	0	0	0	0	0	0	0	-
1977								0	0	0	0	0	0	0	0	0	0	0	0	0	0	-
1978									0	0	0	0	0	0	0	0	0	0	0	0	0	-
1979										0	0	0	0	0	1	0	0	0	0	0	0	-
1980											0	0	0	0	0	0	0	0	0	0	0	-
1981												0	0	0	0	0	0	0	0	0	0	-
1982													0	0	0	0	0	0	0	0	1	-
1983														0	0	0	0	0	0	0	0	-
1984															0	2	0	0	0	0	0	-
1985																0	0	0	0	0	0	-
1986																	2	2	0	0	1	-
1987																		0	1	0	0	-
1988																			0	0	0	-
1989																				0	0	-
1990																					1	-
**Ringed as adults**																						
1970	1	0	0	0	0	0	0	0	0	0	0	0	0	0	0	0	0	0	0	0	0	-
1971		0	0	0	0	0	0	0	0	0	0	0	0	0	0	0	0	0	0	0	0	-
1972			0	0	0	0	0	0	0	0	0	0	0	0	0	0	0	0	0	0	0	-
1973				0	0	0	0	0	0	0	0	0	0	0	0	0	0	0	0	0	0	-
1974					0	0	0	0	0	0	0	0	0	0	0	0	0	0	0	0	0	-
1975						0	0	0	0	0	0	0	0	0	0	0	0	0	0	0	0	-
1976							0	0	0	0	0	0	0	0	0	0	0	0	0	0	0	-
1977								0	0	0	0	0	0	0	0	0	0	0	0	0	0	-
1978									0	0	0	0	0	0	0	0	0	0	0	0	0	-
1979										0	0	1	0	0	0	0	0	0	0	0	0	-
1980											0	0	0	1	0	0	1	0	0	0	0	-
1981												1	0	0	0	0	0	0	1	0	0	-
1982													0	0	0	0	1	0	2	0	0	-
1983														0	1	0	1	0	0	0	0	-
1984															1	0	0	0	0	0	0	-
1985																0	0	0	0	0	0	-
1986																	8	1	0	0	1	-
1987																		0	2	0	0	-
1988																			0	0	0	-
1989																				0	0	-
1990																					1	-

Table S5: Total number of fledged Sandwich Terns ringed from 1970-1990.

Year	1970	1971	1972	1973	1974	1975	1976	1977	1978	1979	1980	1981	1982	1983	1984	1985	1986	1987	1988	1989	1990
	19	11	2	3	10	7	10	32	16	86	19	95	75	87	45	34	301	108	13	97	174

S3 - Multi-Event Model Format

Lebreton (1999) showed that ring-recovery models can be written as a multi-state model by considering two states: alive and dead. Gauthier and Lebreton (2008) show how ring-recovery models can be written in the multistate format used in the program M-Surge (Choquet *et al.*, 2004). To allow for uncertainty in multistates capture-recapture models the multievent frame work was developed by Pradel (2005). Multi-event models can be fitted in the program E-Surge (Choquet *et al.*, 2009). Here we show how the historic model can be written in multievent format.

A multievent model has N states. The multievent format uses data in an encounter history format. An individual animal has history $h = o_1 o_2 o_3 ... o_T$, where o_t is the state at time t with $o_t = 0$ indicating that the individual was not observed. For example $h = 0102$ is an individual that was first observed in state 1 at time 2, was not observed at time 3 and was observed in state 2 at time 4. The multievent model can be summarised using 4 matrices:

- $\mathbf{\Pi}_t$ - a vector of initial state probabilities at time t,

- $\mathbf{\Phi}_t$ - a matrix specifying transitions between states at time t,

- $\mathbf{B}_t$ - a matrix of event probabilities at time t,

- $\mathbf{B}_0$ - a matrix of event probabilities at time 0.

The probability of history h occurring can then be written as

$$\text{Prob}(h) = \mathbf{\Pi}_e \text{diag}\left\{\mathbf{B}_0(\nu_e, .)\right\} \left[\prod_{t=e+1}^{T} \mathbf{\Phi}_{t-1} \text{diag}\left\{\mathbf{B}_t(\nu_t, .)\right\}\right] \mathbf{1}_N,$$

where e is the first encounter of the individual, and where ν_t is the event observed at time t. The vector $\mathbf{B}_t(\nu_t, .)$ is the row vector of $\mathbf{B}$ corresponding to event ν_t, similarly $\mathbf{B}^0(\nu_t, .)$ is the row vector of $\mathbf{B}_0$ corresponding to event ν_t and $\mathbf{1}_N$ is a column vector consisting of N ones. diag $\{\mathbf{V}\}$ is notation used to represent forming a diagonal matrix from vector $\mathbf{V}$. If there are n independent individual histories the likelihood is then

$$L = \prod_{h=1}^{n} \text{Prob}(h)$$

To be able to use the multievent framework we have 3 states observed as juvenile (represented by 1), observed as adult (represented as 2), observed at an unknown age (represented as 3). Note that the first observation is the ringing and the second observation is the recovery all other entries are zeros in a history. If an animal is of an unknown age it is only ever ringed and never recovered (i.e. a 3 is always followed by zeros). As in ring-recovery a bird can be recovered in the year ringed therefore the recovery is recorded in the history the year after. For example the history $h = 1100$ corresponds to a bird ringed as a juvenile in year 1 and recovered dead during year 1, when they were still a juvenile and the history $h = 1020$ corresponds to a bird ringed as a juvenile in year 1 and recovered dead in year 2, but is now an adult. Table S6 gives an example set of data in the m-array format that has been used in this paper compared alongside the equivalent histories.

Table S6: An example comparing m-array format used in this paper with the history format for the data. In the history format no. is the number of histories with that format. 0 represents unobserved, 1 represents observed as a juvenile, 2 represents observed as an adult (the first 1 or 2 corresponds to the ringing and the second 1 corresponds to the recovery) and 3 represents observed at an unknown age (note this is only ever a ringing followed by a string of zeros). Note than unlike capture-recapture every history is not possible. The number of histories observed for the history 3000 is calculated as 8429 − 114 − 39 − 17 − 39 − 18 − 19, etc.

m-array format:

Ringed as juveniles				Ringed as adult				Juv. & adult
Year of ringing	Year of recovery 1	2	3	Year of ringing	Year of recovery 1	2	3	Total ringed
1	114	39	17	1	39	18	19	8429
2		93	26	2		44	23	8401
3			70	3			32	7704

History format:

history	no.	history	no.	history	no.
1100	114	2200	39	3000	8183
1020	39	2020	18		
1002	17	2002	19		
0110	93	0220	44	0300	8215
0102	26	0202	23		
0011	70	0022	32	0030	7602

The matrices for the multievent format os the historic model are then:

$$\mathbf{\Pi}_t = \begin{bmatrix} \pi_t & 1-\pi_t & 0 & 0 & 0 \end{bmatrix},$$

$$\mathbf{\Phi}_t = \begin{bmatrix} 0 & \phi_{1,t} & \phi_{1,t} & 0 & 0 \\ 0 & \phi_{a,t} & 0 & \phi_{a,t} & 0 \\ 0 & 0 & 0 & 0 & 1 \\ 0 & 0 & 0 & 0 & 1 \\ 0 & 0 & 0 & 0 & 1 \end{bmatrix},$$

$$\mathbf{B}_t = \begin{bmatrix} 1 & 1 & 1-\lambda_{1,t-1} & 1-\lambda_{a,t-1} & 1 \\ 0 & 0 & \lambda_{1,t-1} & 0 & 0 \\ 0 & 0 & 0 & \lambda_{a,t-1} & 0 \\ 0 & 0 & 0 & 0 & 0 \end{bmatrix}$$

and

$$\mathbf{B}_0 = \begin{bmatrix} 0 & 0 & 0 & 0 & 1 \\ 1 & 0 & 0 & 0 & 0 \\ 0 & 1 & 0 & 0 & 0 \\ 1 & 1 & 0 & 0 & 0 \end{bmatrix}.$$

The matrices have five states, alive as a juvenile, alive as an adult, died as a juvenile, died as an adult, permanently removed from the population. (The last state is needed as the animal can only be recovered once, Gauthier and Lebreton, 2008). The event matrix, $\mathbf{B}_t$ first row corresponds to the event not observed, the second row corresponds to the event observed as a juvenile, the third row corresponds to observed as an adult and the final row to observed at unknown age.

S4 - Simulation Study for Populations with Homogeneous Survival Probabilities

Simulation results for five, and 20 years of ring-recovery study for an homogeneous population. Results for 10 years of simulation study are presented in the results section of the main paper. We also explain in this section how the length of the study affects the estimation of the parameters for each of the models. In each simulation 100 data sets with constant probabilities ϕ_1, ϕ_a, and λ are simulated from the standard combined model with a total of 1000 birds ringed, with a constant proportion π of these birds ringed as young and $(1-\pi)$ of the birds ringed as adults. We fit a model with separate constant survival probabilities for first year and adult birds, ϕ_1, and ϕ_a respectively, and a constant reporting probability, λ.

Table S7: Simulation study for 20 years of ring-recovery study. The first column specifies the type of model fitted, with Stand. Comb. short for standard combined, and Hist. short for historical. In this first column, the last last two rows contain information for the models in which the proportion parameter was fixed and the values used. The remaining columns contain the average parameter estimate (par est) and the average standard error, given in parentheses, along with the mean squared error (MSE).

	ϕ_1		ϕ_a		λ		π	
	par est	MSE	par est	MSE	par est	MSE	par est	MSE
True Value	0.50	-	0.60	-	0.05	-	0.40	-
Stand. Comb.	0.50(0.03)	0.0006	0.60(0.01)	0.0002	0.05(0.002)	0.0000	-	-
Hist.	0.50(0.03)	0.0006	0.60(0.01)	0.0002	0.05(0.002)	0.0000	0.40(0.02)	0.0002
Hist. $\pi = 0.40$	0.50(0.03)	0.0006	0.60(0.01)	0.0002	0.05(0.002)	0.0000	-	-
Hist. $\pi = 0.20$	0.48(0.04)	0.0008	0.61(0.02)	0.0002	0.05(0.003)	0.0000	-	-

Table S8: Simulation study for five years of ring-recovery study. The first column specifies the type of model fitted, with Stand. Comb. short for standard combined, and Hist. short for historical. In this first column, the last last two rows contain information for the models in which the proportion parameter was fixed and the values used. The remaining columns contain the average parameter estimate (par est) and the average standard error, given in parentheses, along with the mean squared error (MSE).

	ϕ_1		ϕ_a		λ		π	
	par est	MSE	par est	MSE	par est	MSE	par est	MSE
True Value	0.50	-	0.60	-	0.05	-	0.40	-
Stand. Comb.	0.50(0.06)	0.0041	0.60(0.05)	0.0029	0.05(0.001)	0.0000	-	-
Hist.	0.50(0.06)	0.0039	0.60(0.05)	0.0030	0.05(0.005)	0.0000	0.40(0.02)	0.0006
Hist. $\pi = 0.40$	0.50(0.06)	0.0041	0.60(0.06)	0.0030	0.05(0.005)	0.0000	-	-
Hist. $\pi = 0.20$	0.63(0.11)	0.0541	0.81(0.07)	0.0673	0.05(0.200)	0.1432	-	-

S5 - Simulation Study for Populations with Heterogeneous Adult Survival Probability

Simulation results for five, 10, and 20 years of ring-recovery study for heterogeneous populations in terms of adult survival. For these studies, we fit a model with separate constant survival probabilities for first year and adult birds, ϕ_1, and ϕ_a respectively, and a constant reporting probability, λ.

We provide two different types of heterogeneous populations, first we look at a population in which adult survival $\phi_{a,i}$, varies individually, where i denotes a logit-normal individual random effect. We also simulate data for a population formed by two different sub-populations with two different adult survival probabilities $\phi_{a,A}$, and $\phi_{a,B}$.

Simulation Study with Individual Variation in the Adult Survival Probability

In each simulation 100 data sets are simulated from the standard combined model with 1000 birds ringed, with a constant proportion π of these birds ringed as young and $(1-\pi)$ of the birds ringed as adults. To simulate these data, we keep the first year survival probability, ϕ_1, and the reporting probability, λ constant. The adult survival probability, $\phi_{a,i}$, varies according to a logit-normal individual random effect i; $\text{logit}(\phi_{a,i}) = \alpha + \epsilon_i$, with $\epsilon_i \sim N(0, \sigma^2)$. The true value of alpha is set to 0.405, and the mean value of $\phi_{a,i}$ is 0.6. We also set σ^2 to two different values. In Tables (S9, S11, S13) we show the results of using a logit-normal individual random effect with $\sigma^2 = 0.5$, and in Tables (S10, S12, S14) we show the results of setting σ^2 to 1.

Table S9: Simulation study for 20 years of ring-recovery study for a population with heterogeneous adult survival, where the adult survival probability, $\phi_{a,i}$ changes per individual and $\sigma^2 = 0.5$. The first column specifies the type of model fitted, with Stand. Comb. short for standard combined, and Hist. short for historical. In this first column, the last last two rows contain information for the models in which the proportion parameter was fixed and the values used. The remaining columns contain the average parameter estimate (par est) and the average standard error, given in parentheses, along with the mean squared error (MSE).

	ϕ_1		$\phi_{a,i}$		λ		π	
	par est	MSE	par est	MSE	par est	MSE	par est	MSE
True Value	0.50	-	0.6	-	0.05	-	0.40	-
Stand. Comb.	0.50(0.06)	0.0006	0.60(0.01)	0.0002	0.05(0.002)	0.0000	-	-
Hist.	0.50(0.06)	0.0006	0.60(0.01)	0.0002	0.05(0.002)	0.0000	0.40(0.02)	0.0002
Hist. $\pi = 0.40$	0.50(0.06)	0.0006	0.60(0.01)	0.0002	0.05(0.002)	0.0000	-	-
Hist. $\pi = 0.20$	0.48(0.09)	0.0010	0.61(0.01)	0.0003	0.05(0.002)	0.0000	-	-

Table S10: Simulation study for 20 years of ring-recovery study for a population with heterogeneous adult survival, where the adult survival probability, $\phi_{a,i}$ changes per individual and $\sigma^2 = 1$. The first column specifies the type of model fitted, with Stand. Comb. short for standard combined, and Hist. short for historical. In this first column, the last last two rows contain information for the models in which the proportion parameter was fixed and the values used. The remaining columns contain the average parameter estimate (par est) and the average standard error, given in parentheses, along with the mean squared error (MSE).

	ϕ_1		$\phi_{a,i}$		λ		π	
	par est	MSE	par est	MSE	par est	MSE	par est	MSE
True Value	0.50	-	0.6	-	0.05	-	0.40	-
Stand. Comb.	0.49(0.03)	0.0009	0.62(0.01)	0.0008	0.05(0.002)	0.0000	-	-
Hist.	0.49(0.03)	0.0009	0.62(0.01)	0.0008	0.05(0.002)	0.0000	0.40(0.02)	0.0003
Hist. $\pi = 0.40$	0.49(0.03)	0.0009	0.62(0.01)	0.0008	0.05(0.002)	0.0000	-	-
Hist. $\pi = 0.20$	0.48(0.03)	0.0014	0.63(0.01)	0.0011	0.05(0.002)	0.0000	-	-

Table S11: Simulation study for 10 years of ring-recovery study for a population with heterogeneous adult survival, where the adult survival probability, $\phi_{a,i}$ changes per individual and $\sigma^2 = 0.5$. The first column specifies the type of model fitted, with Stand. Comb. short for standard combined, and Hist. short for historical. In this first column, the last last two rows contain information for the models in which the proportion parameter was fixed and the values used. The remaining columns contain the average parameter estimate (par est) and the average standard error, given in parentheses, along with the mean squared error (MSE).

	ϕ_1		$\phi_{a,i}$		λ		π	
	par est	MSE	par est	MSE	par est	MSE	par est	MSE
True Value	0.50	-	0.6	-	0.05	-	0.40	-
Stand. Comb.	0.49(0.04)	0.0015	0.60(0.02)	0.0004	0.05(0.002)	0.0000	-	-
Hist.	0.49(0.04)	0.0015	0.60(0.02)	0.0004	0.05(0.002)	0.0000	0.40(0.02)	0.0006
Hist. $\pi = 0.40$	0.49(0.04)	0.0016	0.60(0.02)	0.0004	0.05(0.002)	0.0000	-	-
Hist. $\pi = 0.20$	0.46(0.04)	0.0032	0.62(0.03)	0.0011	0.05(0.003)	0.0000	-	-

Table S12: Simulation study for 10 years of ring-recovery study for a population with heterogeneous adult survival, where the adult survival probability, $\phi_{a,i}$ changes per individual and $\sigma^2 = 1$. The first column specifies the type of model fitted, with Stand. Comb. short for standard combined, and Hist. short for historical. In this first column, the last last two rows contain information for the models in which the proportion parameter was fixed and the values used. The remaining columns contain the average parameter estimate (par est) and the average standard error, given in parentheses, along with the mean squared error (MSE).

	ϕ_1		$\phi_{a,i}$		λ		π	
	par est	MSE	par est	MSE	par est	MSE	par est	MSE
True Value	0.50	-	0.6	-	0.05	-	0.40	-
Stand. Comb.	0.48(0.04)	0.0024	0.58(0.03)	0.0011	0.05(0.002)	0.0000	-	-
Hist.	0.48(0.04)	0.0023	0.58(0.03)	0.0011	0.05(0.002)	0.0000	0.40(0.02)	0.0006
Hist. $\pi = 0.40$	0.48(0.04)	0.0024	0.58(0.03)	0.0011	0.05(0.002)	0.0000	-	-
Hist. $\pi = 0.20$	0.45(0.04)	0.0048	0.61(0.03)	0.0009	0.05(0.002)	0.0000	-	-

Table S13: Simulation study for five years of ring-recovery study for a population with heterogeneous adult survival, where the adult survival probability, $\phi_{a,i}$ changes per individual and $\sigma^2 = 0.5$. The first column specifies the type of model fitted, with Stand. Comb. short for standard combined, and Hist. short for historical. In this first column, the last last two rows contain information for the models in which the proportion parameter was fixed and the values used. The remaining columns contain the average parameter estimate (par est) and the average standard error, given in parentheses, along with the mean squared error (MSE).

	ϕ_1		$\phi_{a,i}$		λ		π	
	par est	MSE	par est	MSE	par est	MSE	par est	MSE
True Value	0.50	-	0.6	-	0.05	-	0.40	-
Stand. Comb.	0.48(0.06)	0.0040	0.58(0.06)	0.0038	0.05(0.005)	0.0000	-	-
Hist.	0.48(0.06)	0.0041	0.58(0.06)	0.0037	0.05(0.005)	0.0000	0.40(0.04)	0.0012
Hist. $\pi = 0.40$	0.49(0.06)	0.0040	0.58(0.06)	0.0037	0.05(0.005)	0.0000	-	-
Hist. $\pi = 0.20$	0.55(0.10)	0.0319	0.75(0.07)	0.0468	0.16(0.089)	0.0780	-	-

Table S14: Simulation study for five years of ring-recovery study for a population with heterogeneous adult survival, where the adult survival probability, $\phi_{a,i}$ changes per individual and $\sigma^2 = 1$. The first column specifies the type of model fitted, with Stand. Comb. short for standard combined, and Hist. short for historical. In this first column, the last last two rows contain information for the models in which the proportion parameter was fixed and the values used. The remaining columns contain the average parameter estimate (par est) and the average standard error, given in parentheses, along with the mean squared error (MSE).

	ϕ_1		$\phi_{a,i}$		λ		π	
	par est	MSE	par est	MSE	par est	MSE	par est	MSE
True Value	0.50	-	0.6	-	0.05	-	0.40	-
Stand. Comb.	0.46(0.06)	0.0046	0.53(0.05)	0.0086	0.05(0.004)	0.0000	-	-
Hist.	0.46(0.06)	0.0048	0.53(0.05)	0.0090	0.05(0.004)	0.0000	0.41(0.04)	0.0018
Hist. $\pi = 0.40$	0.46(0.06)	0.0046	0.53(0.05)	0.0086	0.05(0.004)	0.0000	-	-
Hist. $\pi = 0.20$	0.51(0.09)	0.0328	0.68(0.07)	0.0388	0.05(0.099)	0.0068	-	-

Simulation Study for two Populations with Different Constant Adult Survival Probabilities

For each study we generate data for two sub-populations that have the same constant first year probability ϕ_1, and reporting probability λ, but a different constant adult survival probability. Then we merge these data together into a data set of 2000 ringed birds with heterogeneous adult survival. We simulate two sets of 100 data sets from the standard combined model with 1000 birds ringed in each set, with a similar constant proportion π of these birds ringed as young and $(1 - \pi)$ of the birds ringed as adults. Each of these two sets of 100 data sets, have the same constant first year survival probability, and the same reporting probability, but each of them have a different constant adult survival probability ϕ_a.

We provide results for five, 10 and 20 years of ring-recovery studies, and three case scenarios in which the two sub-populations have different adult survival probabilities. The first population is formed by two sub-populations with adult survival probabilities of 0.4 and 0.7 respectively, the second has two adult survival probabilities of 0.4 and 0.8, and finally the last heterogeneous population has adult survival probabilities of 0.3 and 0.8 for each sub-population.

Table S15: Simulation study for 20 years of ring-recovery study for a population with heterogeneous adult survival, where there are two different adult survival probabilities, $\phi_{a,A} = 0.4$ and $\phi_{a,B} = 0.7$. The first column specifies the type of model fitted, The first column specifies the type of model, with Stand. Comb. short for standard combined, and Hist. short for historical. In this first column, the last last two rows contain information for the models in which the proportion parameter was fixed and the values used. The remaining columns contain the average parameter estimate (par est) and the average standard error, given in parentheses, along with the mean squared error (MSE).

	ϕ_1		$\phi_{a,A}, \phi_{a,B}$	λ		π	
	par est	MSE	par est	par est	MSE	par est	MSE
True Value	0.50	-	0.40, 0.70	0.05	-	0.40	-
Stand. Comb.	0.50(0.02)	0.0003	0.58(0.01)	0.05(0.001)	0.0000	-	-
Hist.	0.50(0.02)	0.0003	0.58(0.01)	0.05(0.001)	0.0000	0.40(0.01)	0.0001
Hist. $\pi = 0.40$	0.50(0.02)	0.0004	0.58(0.01)	0.05(0.001)	0.0000	-	-
Hist. $\pi = 0.20$	0.49(0.02)	0.0006	0.59(0.01)	0.05(0.001)	0.0000	-	-

Table S16: Simulation study for 20 years of ring-recovery study for a population with heterogeneous adult survival, where there are two different adult survival probabilities, $\phi_{a,A} = 0.4$ and $\phi_{a,B} = 0.8$. The first column specifies the type of model fitted, with Stand. Comb. short for standard combined, and Hist. short for historical. In this first column, the last last two rows contain information for the models in which the proportion parameter was fixed and the values used. The remaining columns contain the average parameter estimate (par est) and the average standard error, given in parentheses, along with the mean squared error (MSE).

	ϕ_1		$\phi_{a,A}, \phi_{a,B}$	λ		π	
	par est	MSE	par est	par est	MSE	par est	MSE
True Value	0.50	-	0.40, 0.80	0.05	-	0.40	-
Stand. Comb.	0.49(0.02)	0.0004	0.66(0.01)	0.05(0.001)	0.0000	-	-
Hist.	0.49(0.02)	0.0004	0.66(0.01)	0.05(0.001)	0.0000	0.40(0.01)	0.0001
Hist. $\pi = 0.40$	0.49(0.02)	0.0004	0.66(0.01)	0.05(0.001)	0.0000	-	-
Hist. $\pi = 0.20$	0.47(0.02)	0.0010	0.66(0.01)	0.05(0.001)	0.0000	-	-

Table S17: Simulation study for 20 years of ring-recovery study for a population with heterogeneous adult survival, where there are two different adult survival probabilities, $\phi_{a,A} = 0.3$ and $\phi_{a,B} = 0.8$. The first column specifies the type of model fitted, with Stand. Comb. short for standard combined, and Hist. short for historical. In this first column, the last last two rows contain information for the models in which the proportion parameter was fixed and the values used. The remaining columns contain the average parameter estimate (par est) and the average standard error, given in parentheses, along with the mean squared error (MSE).

	ϕ_1		$\phi_{a,A}, \phi_{a,B}$	λ		π	
	par est	MSE	par est	par est	MSE	par est	MSE
True Value	0.50	-	0.30, 0.80	0.05	-	0.40	-
Stand. Comb.	0.49(0.02)	0.0004	0.64(0.01)	0.05(0.001)	0.0000	-	-
Hist.	0.49(0.02)	0.0004	0.64(0.01)	0.05(0.001)	0.0000	0.40(0.01)	0.0001
Hist. $\pi = 0.40$	0.49(0.02)	0.0004	0.64(0.01)	0.05(0.001)	0.0000	-	-
Hist. $\pi = 0.20$	0.48(0.02)	0.0008	0.64(0.01)	0.05(0.001)	0.0000	-	-

Table S18: Simulation study for 10 years of ring-recovery study study for a population with heterogeneous adult survival, where there are two different adult survival probabilities, $\phi_{a,A} = 0.4$ and $\phi_{a,B} = 0.7$. The first column specifies the type of model fitted, with Stand. Comb. short for standard combined, and Hist. short for historical. In this first column, the last last two rows contain information for the models in which the proportion parameter was fixed and the values used. The remaining columns contain the average parameter estimate (par est) and the average standard error, given in parentheses, along with the mean squared error (MSE).

	ϕ_1		$\phi_{a,A}, \phi_{a,B}$	λ		π	
	par est	MSE	par est	par est	MSE	par est	MSE
True Value	0.50	-	0.40,0.70	0.05	-	0.40	-
Stand. Comb.	0.50(0.03)	0.0008	0.56(0.02)	0.05(0.002)	0.0000	-	-
Hist.	0.50(0.03)	0.0008	0.56(0.02)	0.05(0.002)	0.0000	0.40(0.02)	0.0004
Hist. $\pi = 0.40$	0.50(0.03)	0.0007	0.56(0.02)	0.05(0.002)	0.0000	-	-
Hist. $\pi = 0.20$	0.47(0.03)	0.0007	0.58(0.02)	0.05(0.002)	0.0000	-	-

Table S19: Simulation study for 10 years of ring-recovery study for a population with heterogeneous adult survival, where there are two different adult survival probabilities, $\phi_{a,A} = 0.4$ and $\phi_{a,B} = 0.8$. The first column specifies the type of model fitted, with Stand. Comb. short for standard combined, and Hist. short for historical. In this first column, the last last two rows contain information for the models in which the proportion parameter was fixed and the values used. The remaining columns contain the average parameter estimate (par est) and the average standard error, given in parentheses, along with the mean squared error (MSE).

	ϕ_1		$\phi_{a,A}, \phi_{a,B}$	λ		π	
	par est	MSE	par est	par est	MSE	par est	MSE
True Value	0.50	-	0.40,0.80	0.05	-	0.40	-
Stand. Comb.	0.48(0.03)	0.0010	0.60(0.02)	0.05(0.002)	0.0000	-	-
Hist.	0.48(0.03)	0.0010	0.60(0.02)	0.05(0.002)	0.0000	0.41(0.02)	0.0005
Hist. $\pi = 0.40$	0.48(0.03)	0.0010	0.61(0.02)	0.05(0.002)	0.0000	-	-
Hist. $\pi = 0.20$	0.45(0.03)	0.0033	0.63(0.02)	0.05(0.002)	0.0000	-	-

Table S20: Simulation study for 10 years of ring-recovery study for a population with heterogeneous adult survival, where there are two different adult survival probabilities, $\phi_{a,A} = 0.3$ and $\phi_{a,B} = 0.8$. The first column specifies the type of model fitted, with Stand. Comb. short for standard combined, and Hist. short for historical. In this first column, the last last two rows contain information for the models in which the proportion parameter was fixed and the values used. The remaining columns contain the average parameter estimate (par est) and the average standard error, given in parentheses, along with the mean squared error (MSE).

	ϕ_1		$\phi_{a,A}, \phi_{a,B}$	λ		π	
	par est	MSE	par est	par est	MSE	par est	MSE
True Value	0.50	-	0.30,0.80	0.05	-	0.40	-
Stand. Comb.	0.48(0.03)	0.0013	0.57(0.02)	0.05(0.002)	0.0000	-	-
Hist.	0.48(0.03)	0.0013	0.57(0.02)	0.05(0.002)	0.0000	0.41(0.02)	0.0004
Hist. $\pi = 0.40$	0.48(0.03)	0.0014	0.57(0.02)	0.05(0.002)	0.0000	-	-
Hist. $\pi = 0.20$	0.44(0.03)	0.0039	0.58(0.02)	0.05(0.002)	0.0000	-	-

Table S21: Simulation study for five years of ring-recovery study for a population with heterogeneous adult survival, where there are two different adult survival probabilities, $\phi_{a,A} = 0.4$ and $\phi_{a,B} = 0.7$. The first column specifies the type of model fitted, with Stand. Comb. short for standard combined, and Hist. short for historical. In this first column, the last last two rows contain information for the models in which the proportion parameter was fixed and the values used. The remaining columns contain the average parameter estimate (par est) and the average standard error, given in parentheses, along with the mean squared error (MSE).

	ϕ_1		$\phi_{a,A}, \phi_{a,B}$	λ		π	
	par est	MSE	par est	par est	MSE	par est	MSE
True Value	0.50	-	0.40,0.70	0.05	-	0.40	-
Stand. Comb.	0.48(0.04)	0.0021	0.53(0.04)	0.05(0.003)	0.0000	-	-
Hist.	0.48(0.04)	0.0021	0.53(0.04)	0.05(0.003)	0.0000	0.40(0.02)	0.0008
Hist. $\pi = 0.40$	0.48(0.04)	0.0021	0.53(0.04)	0.05(0.003)	0.0000	-	-
Hist. $\pi = 0.20$	0.45(0.05)	0.0077	0.63(0.05)	0.06(0.017)	0.0031	-	-

Table S22: Simulation study for five years of ring-recovery study study for a population with heterogeneous adult survival, where there are two different adult survival probabilities, $\phi_{a,A}$ and $\phi_{a,B}$. The first column specifies the type of model fitted, with Stand. Comb. short for standard combined, and Hist. short for historical. In this first column, the last last two rows contain information for the models in which the proportion parameter was fixed and the values used. The remaining columns contain the average parameter estimate (par est) and the average standard error, given in parentheses, along with the mean squared error (MSE).

	ϕ_1		$\phi_{a,A}, \phi_{a,B}$	λ		π	
	par est	MSE	par est	par est	MSE	par est	MSE
Stand. Comb.	0.46(0.04)	0.0031	0.55(0.04)	0.05(0.003)	0.0000	-	-
Hist.	0.47(0.04)	0.0028	0.54(0.04)	0.05(0.003)	0.0000	0.42(0.03)	0.0011
Hist. $\pi = 0.40$	0.46(0.04)	0.0030	0.55(0.04)	0.05(0.003)	0.0000	-	-
Hist. $\pi = 0.20$	0.50(0.08)	0.0237	0.70(0.07)	0.11(0.06)	0.0385	-	-

Table S23: Simulation study for five years of ring-recovery study for a population with heterogeneous adult survival, where there are two different adult survival probabilities, $\phi_{a,A}$ and $\phi_{a,B}$. The first column specifies the type of model fitted, with Stand. Comb. short for standard combined, and Hist. short for historical. In this first column, the last last two rows contain information for the models in which the proportion parameter was fixed and the values used. The remaining columns contain the average parameter estimate (par est) and the average standard error, given in parentheses, along with the mean squared error (MSE).

	ϕ_1		$\phi_{a,A}, \phi_{a,B}$	λ		π	
	par est	MSE	par est	par est	MSE	par est	MSE
True Value	0.50	-	0.30,0.80	0.05	-	0.40	-
Stand. Comb.	0.45(0.04)	0.0046	0.49(0.04)	0.04(0.003)	0.0000	-	-
Hist.	0.46(0.04)	0.0042	0.49(0.04)	0.04(0.003)	0.0000	0.42(0.03)	0.0010
Hist. $\pi = 0.40$	0.45(0.04)	0.0046	0.49(0.05)	0.04(0.003)	0.0000	-	-
Hist. $\pi = 0.20$	0.42(0.05)	0.0139	0.58(0.05)	0.06(0.017)	0.0120	-	-

S6 - Sandwich Tern Results

Figures S1 and S2 show the time dependent parameters for two of the best historical combined data models fitted to the Sandwich Tern data. Figure S1 shows the time dependency

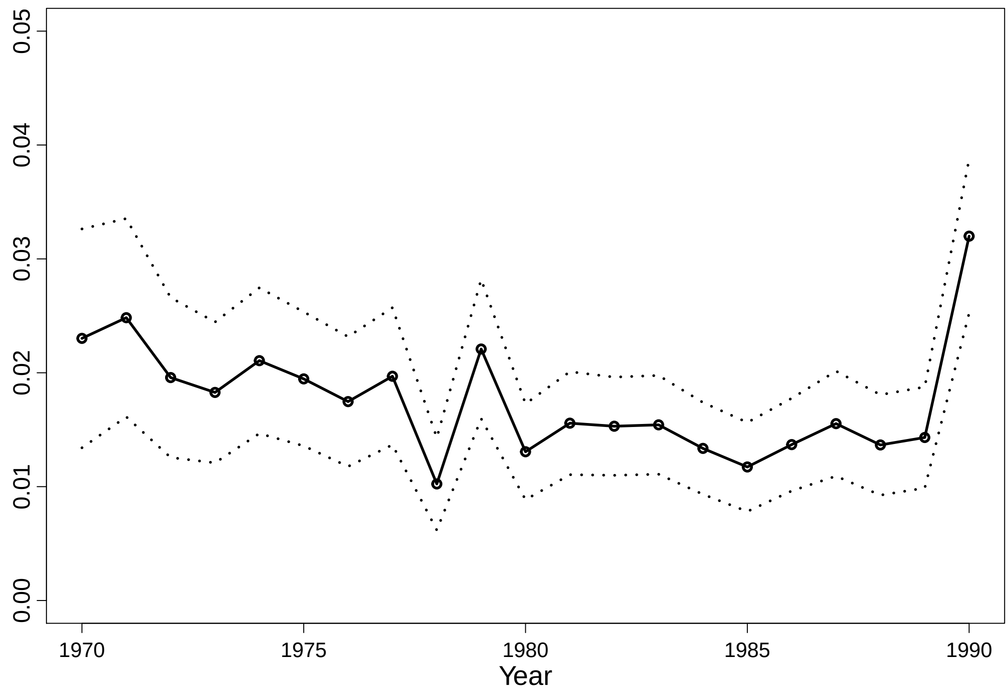

Figure S1: Estimated recovery probability, λ_t, for the historical combined data model with parameters (ϕ_1, ϕ_a, λ_t, π) for Sandwich Tern data for the years 1970-1990. The continuous line represent the estimated recovery probability, and the dotted lines represent 95 % confindece interval.

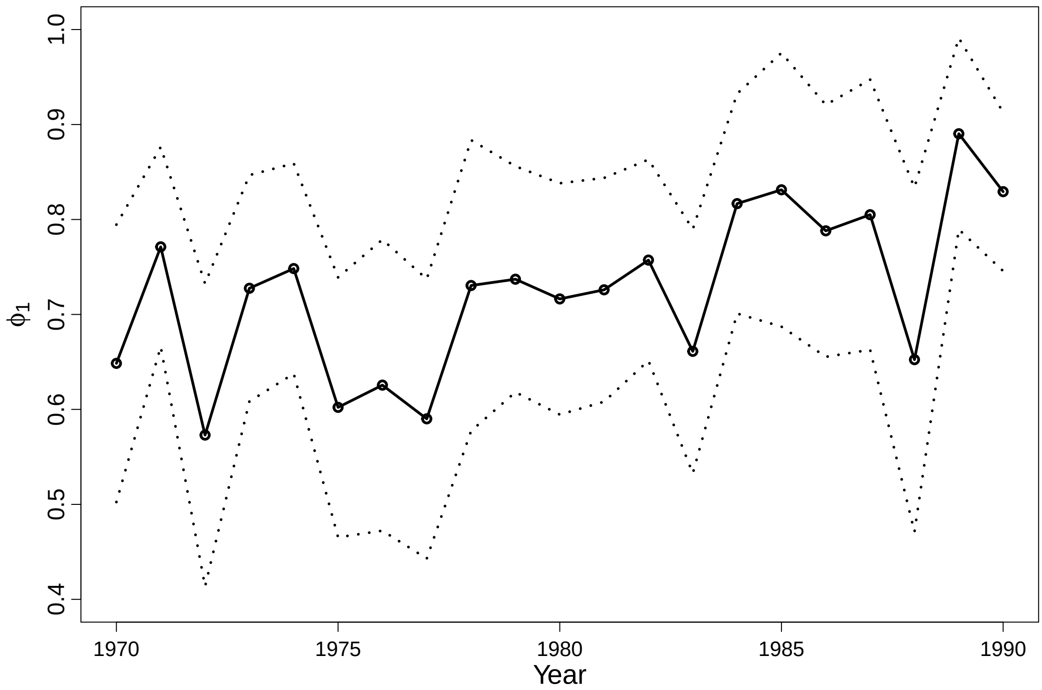

Figure S2: Estimated first year survival probability, $\phi_{1,t}$, for the historical combined data model with parameters ($\phi_{1,t}$, ϕ_a, λ, π) for Sandwich Tern data for the years 1970-1990. The continuous line represent the estimated survival probability, and the dotted lines represent 95 % confindece interval.

S7 - R Code for Historical Combined Data Model

```
## Combined model: General code to fit all models using Blackbird data as example
#constant and time dependent models for:phi1, phia, lambda1 & lambda2
# Example with numbers set for the C/T/T/C model
# Formed by: 1. Standard Model + 2. Historic Model +
# 3. Likelihood for Combined Model + 4. MLE for Combined Model
# Note that with small changes in the code the standard and historic model can be fitted separetely
#####################################################################################
######################## Create expit and logit functions ########################
expit <- function(xval)
{
  1/(1+exp(-xval))
}

logit <- function(xval)
{
  log(xval/(1-xval))
}
#####################################################################################
##################### 1. Standard Model: birds ringed as pulli #####################
#####################################################################################
###### DATA
###### Datamatrix: matrix of recoveries for birds ringed as pulli
Datamatrix <- matrix(c(
  52, 15, 10,  2,   8,   5,  3,  1,  0,  1,  0,  0,  0,  0,  0,  0,  0,  0,  0,  0,
   0, 74, 30, 14,  18,   7, 12,  2,  1,  3,  1,  1,  0,  0,  0,  0,  0,  0,  0,  0,
   0,  0, 78, 29,  20,  11,  4,  4,  2,  0,  2,  1,  0,  1,  0,  0,  0,  0,  0,  0,
   0,  0,  0, 67,  22,  12,  4,  7,  4,  2,  2,  1,  4,  1,  2,  0,  0,  0,  0,  0,
   0,  0,  0,  0, 101,  30, 10,  8,  8,  6,  3,  6,  1,  1,  1,  0,  0,  0,  0,  0,
   0,  0,  0,  0,   0,  81, 27, 10, 11, 10,  4,  1,  3,  1,  3,  1,  0,  0,  1,  0,
   0,  0,  0,  0,   0,   0, 58, 15,  8,  5,  4,  4,  3,  2,  0,  0,  1,  1,  0,  0,
   0,  0,  0,  0,   0,   0,  0, 55, 19, 11, 10,  6,  6,  5,  3,  0,  1,  1,  0,  0,
   0,  0,  0,  0,   0,   0,  0,  0, 67, 21, 14,  6,  7,  6,  1,  0,  2,  2,  0,  1,
   0,  0,  0,  0,   0,   0,  0,  0,  0, 54, 26,  6,  8,  2,  4,  2,  1,  0,  1,  1,
   0,  0,  0,  0,   0,   0,  0,  0,  0,  0, 42, 17,  5,  9,  3,  1,  1,  3,  0,  0,
   0,  0,  0,  0,   0,   0,  0,  0,  0,  0,  0, 57, 12,  8,  4,  2,  5,  0,  0,  1,
   0,  0,  0,  0,   0,   0,  0,  0,  0,  0,  0,  0, 58, 15, 10,  5,  8,  2,  3,  2,
   0,  0,  0,  0,   0,   0,  0,  0,  0,  0,  0,  0,  0, 64, 13,  7,  7,  4,  3,  1,
   0,  0,  0,  0,   0,   0,  0,  0,  0,  0,  0,  0,  0,  0, 60, 21,  8, 10,  9,  1,
   0,  0,  0,  0,   0,   0,  0,  0,  0,  0,  0,  0,  0,  0,  0, 62, 15, 11,  4,  9,
   0,  0,  0,  0,   0,   0,  0,  0,  0,  0,  0,  0,  0,  0,  0,  0, 54, 15,  8,  9,
   0,  0,  0,  0,   0,   0,  0,  0,  0,  0,  0,  0,  0,  0,  0,  0,  0, 57, 18,  8,
   0,  0,  0,  0,   0,   0,  0,  0,  0,  0,  0,  0,  0,  0,  0,  0,  0,  0, 41, 14,
   0,  0,  0,  0,   0,   0,  0,  0,  0,  0,  0,  0,  0,  0,  0,  0,  0,  0,  0, 70
),nrow=20,ncol=20,byrow=TRUE)

n1 <-nrow(Datamatrix) # number of years of ringing
n2 <-ncol(Datamatrix) # number of years of recovery
###### f: Total number of birds never recovered that were ringed as pulli
f <- c
     (2391,3420,4366,4187,4172,4364,3347,3344,3618,3034,2730,3077,3038,3436,3537,3817,3317,3427,2872,4080)

####### DEFINE PARAMETERS NEEDED FOR THE STANDARD MODEL
# Number of rows define the year i.e: time dependency
# Number of columns define the age, i.e: age dependency
# For parameters with time dependency :change the number of pars needed in the model [year1:year21]
pars_fun_ccc <- function(pars_in){
```

```
#If phia is constant use the line below:
# This is because we assume birds ringed as pulli to become adults in their 2nd year of life
  #phia<-matrix(rep(c(0,expit(pars_in[1])),c(1,(n1-1))),n1,n2,byrow=TRUE)
#If phia is time dependent, use the line below (notice phia would start from 2 now):
  phia<-matrix(c(0,expit(pars_in[2:20])),n1,n2,byrow=TRUE)
  phi1<-matrix(expit(pars_in[21]),n1, n2, byrow=TRUE)
#If there is no age dependency in lambda, lambda1 and lambda2 share the same parameter numbers as in this
      example
  lambda1 <-matrix(expit(pars_in[22:41]),n1, n2,byrow=TRUE)
  lambda2 <-matrix(expit(pars_in[22:41]),n1,n2,byrow=TRUE)
#If there is an age dependency, for example in the C/T/CA1:2 model, use:
#lambda1 <-matrix(expit(pars_in[22]),n1, n2,byrow=TRUE)
#lambda2 <-matrix(expit(pars_in[23]),n1,n2,byrow=TRUE)
  ringlik(Datamatrix, phia, phi1, lambda1, lambda2)
}
#################################### Likelihood ####################################
nprob <-rep(1,n1)
ringlik <-function(Datamatrix, phia, phi1, lambda1, lambda2)
{
  lik=0
  for (i in 1:nrow(Datamatrix)){
    for (j in i:ncol(Datamatrix)) {
            if(i==j) {
        prob=(1-phi1[i,j])*lambda1[i,j]
      }
      else{
        if(j > (i+1)) prodphi <- prod(phia[i,(i+1):(j-1)]) else prodphi <- 1
        prob=phi1[i,j]*prodphi*(1-phia[i,j])*lambda2[i,j]}
      nprob[i]=nprob[i]-prob
      lik=lik+Datamatrix[i,j]*log(prob)
    }
    lik=lik+f[i]*log(nprob[i])
  }
  lik=-lik
  return(lik)
}

#pars_in: Starting values for the parameters in the same order: phia, phi1, lambda1, lambda2
pars_in<- logit(rep(c(0.6,0.5,0.01),c(20,1,20)))
pars_fun_ccc(pars_in) # gives likelihood for the standard model

#####################################################################################
################### 2. Historic Model: birds ringed as full-grown ###################
#####################################################################################
###### DATA
###### Datamatrixj: matrix of recoveries for birds ringed as juvenile
###### Datamatrixa: matrix of recoveries for birds ringed as adult
Datamatrixj <-matrix(c(
  114,  39,  17,  12,  10,  12,   8,   5,   3,   3,   0,   0,   0,   0,   0,   0,   0,   0,   0,   0,
    0,  93,  26,  31,  19,  16,   6,   7,   3,   6,   1,   1,   0,   0,   0,   1,   0,   0,   0,   0,
    0,   0,  70,  32,  19,  20,   8,   5,   4,   4,   2,   1,   1,   1,   0,   0,   0,   0,   0,   0,
    0,   0,   0,  85,  34,  21,  25,  13,  11,   4,   2,   4,   3,   0,   2,   0,   0,   0,   0,   0,
    0,   0,   0,   0,  75,  30,  34,  18,  16,   8,  11,   5,   6,   2,   1,   0,   0,   0,   0,   0,
    0,   0,   0,   0,   0,  76,  30,  28,  25,  20,   9,   7,   5,   5,   2,   2,   0,   0,   0,   0,
    0,   0,   0,   0,   0,   0,  61,  27,  23,  21,  13,   6,   7,   4,   3,   2,   0,   0,   0,   0,
    0,   0,   0,   0,   0,   0,   0,  53,  34,  15,  14,  11,  10,   4,   8,   1,   1,   0,   0,   0,
    0,   0,   0,   0,   0,   0,   0,   0,  74,  21,  17,  14,  11,   8,   9,   6,   3,   1,   1,   0,
    0,   0,   0,   0,   0,   0,   0,   0,   0,  81,  28,  25,  19,   14,  13,   9,   6,   5,   2,   2,
```

```
  0,  0,  0,  0,  0,  0,  0,  0,  0,  0, 67, 16, 11, 11, 11, 10,  2,  1,  0,  0,
  0,  0,  0,  0,  0,  0,  0,  0,  0,  0,  0, 71, 33, 17, 15,  7,  6,  1,  5,  0,
  0,  0,  0,  0,  0,  0,  0,  0,  0,  0,  0,  0, 68, 28, 17, 11, 13,  5,  8,  4,
  0,  0,  0,  0,  0,  0,  0,  0,  0,  0,  0,  0,  0, 82, 34, 23, 13,  9,  7,  3,
  0,  0,  0,  0,  0,  0,  0,  0,  0,  0,  0,  0,  0,  0, 74, 30, 13, 15,  8,  7,
  0,  0,  0,  0,  0,  0,  0,  0,  0,  0,  0,  0,  0,  0,  0, 75, 29, 17, 17, 13,
  0,  0,  0,  0,  0,  0,  0,  0,  0,  0,  0,  0,  0,  0,  0,  0, 61, 35, 24, 13,
  0,  0,  0,  0,  0,  0,  0,  0,  0,  0,  0,  0,  0,  0,  0,  0,  0, 62, 31, 22,
  0,  0,  0,  0,  0,  0,  0,  0,  0,  0,  0,  0,  0,  0,  0,  0,  0,  0, 59, 36,
  0,  0,  0,  0,  0,  0,  0,  0,  0,  0,  0,  0,  0,  0,  0,  0,  0,  0,  0, 99
),nrow=20,ncol=20,byrow=TRUE)

Datamatrixa<-matrix(c(
  39, 18, 19,  8,  9,  3,  0,  2,  5,  0,  0,  0,  0,  0,  0,  0,  0,  0,  0,  0,
  0, 44, 23, 16, 15, 16,  4,  6,  1,  1,  0,  0,  1,  0,  0,  0,  0,  0,  0,  0,
  0,  0, 32, 27, 10, 11, 10,  6,  3,  4,  1,  1,  0,  0,  0,  0,  0,  0,  0,  0,
  0,  0,  0, 42, 29, 24, 12, 10,  6,  4,  4,  4,  3,  0,  0,  0,  0,  0,  0,  0,
  0,  0,  0,  0, 38, 31, 15, 14, 11,  4,  6,  5,  1,  0,  0,  0,  0,  0,  0,  0,
  0,  0,  0,  0,  0, 62, 29, 18, 20, 15, 12,  8,  4,  2,  2,  0,  0,  0,  0,  0,
  0,  0,  0,  0,  0,  0, 47, 25, 21, 20, 18,  7,  3,  3,  0,  2,  0,  0,  0,  0,
  0,  0,  0,  0,  0,  0,  0, 32, 39, 20, 15, 11, 13,  3,  2,  4,  0,  0,  0,  0,
  0,  0,  0,  0,  0,  0,  0,  0, 38, 28, 25, 20, 14,  9,  3,  3,  0,  0,  1,  0,
  0,  0,  0,  0,  0,  0,  0,  0,  0, 42, 33, 24, 17, 10,  7,  5,  1,  1,  0,  0,
  0,  0,  0,  0,  0,  0,  0,  0,  0,  0, 35, 28, 26, 16, 11,  3,  6,  2,  0,  0,
  0,  0,  0,  0,  0,  0,  0,  0,  0,  0,  0, 37, 25, 15, 10,  9, 10,  4,  4,  0,
  0,  0,  0,  0,  0,  0,  0,  0,  0,  0,  0,  0, 36, 31, 19, 12,  8,  6,  1,  2,
  0,  0,  0,  0,  0,  0,  0,  0,  0,  0,  0,  0,  0, 41, 23, 17, 15, 11,  2,  2,
  0,  0,  0,  0,  0,  0,  0,  0,  0,  0,  0,  0,  0,  0, 38, 16, 18, 13, 10,  8,
  0,  0,  0,  0,  0,  0,  0,  0,  0,  0,  0,  0,  0,  0,  0, 41, 31, 28,  7,  9,
  0,  0,  0,  0,  0,  0,  0,  0,  0,  0,  0,  0,  0,  0,  0,  0, 50, 38, 23, 16,
  0,  0,  0,  0,  0,  0,  0,  0,  0,  0,  0,  0,  0,  0,  0,  0,  0, 48, 26, 30,
  0,  0,  0,  0,  0,  0,  0,  0,  0,  0,  0,  0,  0,  0,  0,  0,  0,  0, 25, 21,
  0,  0,  0,  0,  0,  0,  0,  0,  0,  0,  0,  0,  0,  0,  0,  0,  0,  0,  0, 41
),nrow=20,ncol=20,byrow=TRUE)

###### fhist: Total number of birds never recovered that were ringed as full-grown birds
fhist <- c(6076,6755,6308,7858,8098,7815,7170,7026,7586,7834,
           6968,8951,8815,9101,8668,8933,10041,10237,9551,11472)

####### DEFINE PARAMETERS NEEDED FOR THE HISTORIC MODEL
# Note that the order for the parameters must be the same than the order used for the standard model
pars_fun_cccc <- function(pars_in){
# Here line for phia is the same when constant or time dependent, as we have information of adults since
# the first year of ringing. Note we still need to change from 1 to 1:20 depending if the model is constant
# or time dependent
  phia<-matrix(expit(pars_in[1:20]),n1, n2, byrow=TRUE)
  phi1<-matrix(expit(pars_in[21]),n1, n2, byrow=TRUE)
  lambda1 <-matrix(expit(pars_in[22:41]),n1, n2,byrow=TRUE)
  lambda2 <-matrix(expit(pars_in[22:41]),n1, n2,byrow=TRUE)
#If there is an age dependency, for example in the C/T/CA1:2/C model, we use:
#lambda1 <-matrix(expit(pars_in[22]),n1, n2,byrow=TRUE)
#lambda2 <-matrix(expit(pars_in[23]),n1,n2,byrow=TRUE)
#then, prop <- matrix(expit(pars_in[24]),n1, n2, byrow=TRUE)
  prop <- matrix(expit(pars_in[42]),n1, n2, byrow=TRUE)
  ringlikhist(Datamatrixj,Datamatrixa, phia, phi1, lambda1, lambda2,prop)
}
####### LIKELIHOOD FOR THE HISTORIC MODEL
```

```
nprob <-rep(1,n1)
ringlikhist <-function( Datamatrixj,Datamatrixa, phia, phi1, lambda1, lambda2, prop)
{
  lik=0
  for (i in 1:nrow(Datamatrixj)){
    for (j in i:ncol(Datamatrixj)) {
      if(j > i) prodphia <- prod(phia[i,i:(j-1)]) else prodphia <- 1
      proba=prodphia*(1-phia[i,j])*lambda2[i,j]*(1-prop[i,j])
      if(i==j) {
        probj=(1-phi1[i,j])*lambda1[i,j]*prop[i,j]
      }
      else{
        if(j > (i+1)) prodphi <- prod(phia[i,(i+1):(j-1)]) else prodphi <- 1
        probj=phi1[i,j]*prodphi*(1-phia[i,j])*lambda2[i,j]*prop[i,j]}
      nprob[i]=nprob[i]-probj-proba
      lik=lik+Datamatrixj[i,j]*log(probj)+Datamatrixa[i,j]*log(proba)
    }
    lik=lik+fhist[i]*log(nprob[i])
  }
  lik=-lik
  return(lik)
}
#pars_in: Starting values for the parameters in the same order: phia, phi1, lambda1, lambda2
pars_in<- logit(rep(c(0.6,0.5,0.01,0.3),c(20,1,20,1)))
pars_fun_cccc(pars_in) # returns the likelihood for the historic model
#####################################################################################
########################### 3. Likelihood for Combined Model ########################
#####################################################################################
ringlikcomb <- function(pars_in) {
  lik= pars_fun_ccc(pars_in)+pars_fun_cccc(pars_in)
  return(lik)
}
pars_in<- logit(rep(c(0.6,0.5,0.01,0.3),c(1,20,20,1)))
ringlikcomb(pars_in) # returns the likelihood for the historic combined model
#####################################################################################
############################## 4. MLE for Combined Model ############################
#####################################################################################
logitpars_fit <- optim(par = pars_in, fn = ringlikcomb,
                       method="L-BFGS-B",hessian=TRUE,
                       control=list(maxit=1000000, trace = 6))

# The MLE for the other models can be easily obtained by  modyfying fn in logitpars_fit
# i.e. MLE for historic model:
#logitpars_fit <- optim(par = pars_in, fn = ringlikhist,
                 #method="L-BFGS-B",hessian=TRUE,
                #control=list(maxit=1000000, trace = 6))

AIC <- logitpars_fit$value*2+2*length(pars_in) # returns AIC value
## Obtain parameter estimates
parsest <- expit(logitpars_fit$par)
parsest # Notice order: phia, phi1, lambda1, lambda2, prop
## Obtain standard errors
se <- sqrt(diag(solve(logitpars_fit$hess)))
## Final standard errors, after transformation applying delta method
se_est<- se*parsest*(1-parsest)
se_est
#Check for parameter redundancy
round(eigen(logitpars_fit$hess)$values/max(eigen(logitpars_fit$hess)$values),5)
```

Bibliography

Choquet, R., Reboulet, A.M., Pradel, R., Gimenez, O. & Lebreton J.D. (2004) M-SURGE: new software specifically designed for multistate capture-recapture models. *Animal Biodiversity and Conservation*, **27**, 207-215.

Choquet, R., Rouan, L. & Pradel, R. (2009) Program E-SURGE: a software application for fitting Multievent models. In: Modeling demographic processes in marked populations. Thomson, D., Cooch, E.G. & Conroy, M.J. (Eds.) Springer series: *Environmental and Ecological Statistics*, **3**, 845-865.

Gauthier, G. & Lebreton, J.D. (2008) Analysis of bandrecovery data in a multistate capture-recapture framework. *Canadian Journal of Statistics*, **36**, 59-73.

Lebreton, J.D., Aimeras, T. & R. Pradel, (1999) Competing events, mixtures of information and multistratum recapture models. *Bird Study*, **46** (Supplement), S39-S46.

Pradel, R. (2005) Multi-event: an extension of multi-state capture-recapture models to uncertain states. *Biometrics*, **61**, 442-447.

Robinson, R.A., Baillie, S.R. & King, R. (2012) Population processes in European Blackbirds *Turdus merula*: a state-space approach. *Journal of Ornithology*, **152**, S419-S433.
