# Supplementary figures and images for: Estimating age‐dependent survival from age‐aggregated ringing data—extending the use of historical records
Source: Ecol Evol. 2019 Feb 5;9(2):769–79. doi: 10.1002/ece3.4820 (PMC6362446; doi:10.1002/ece3.4820)

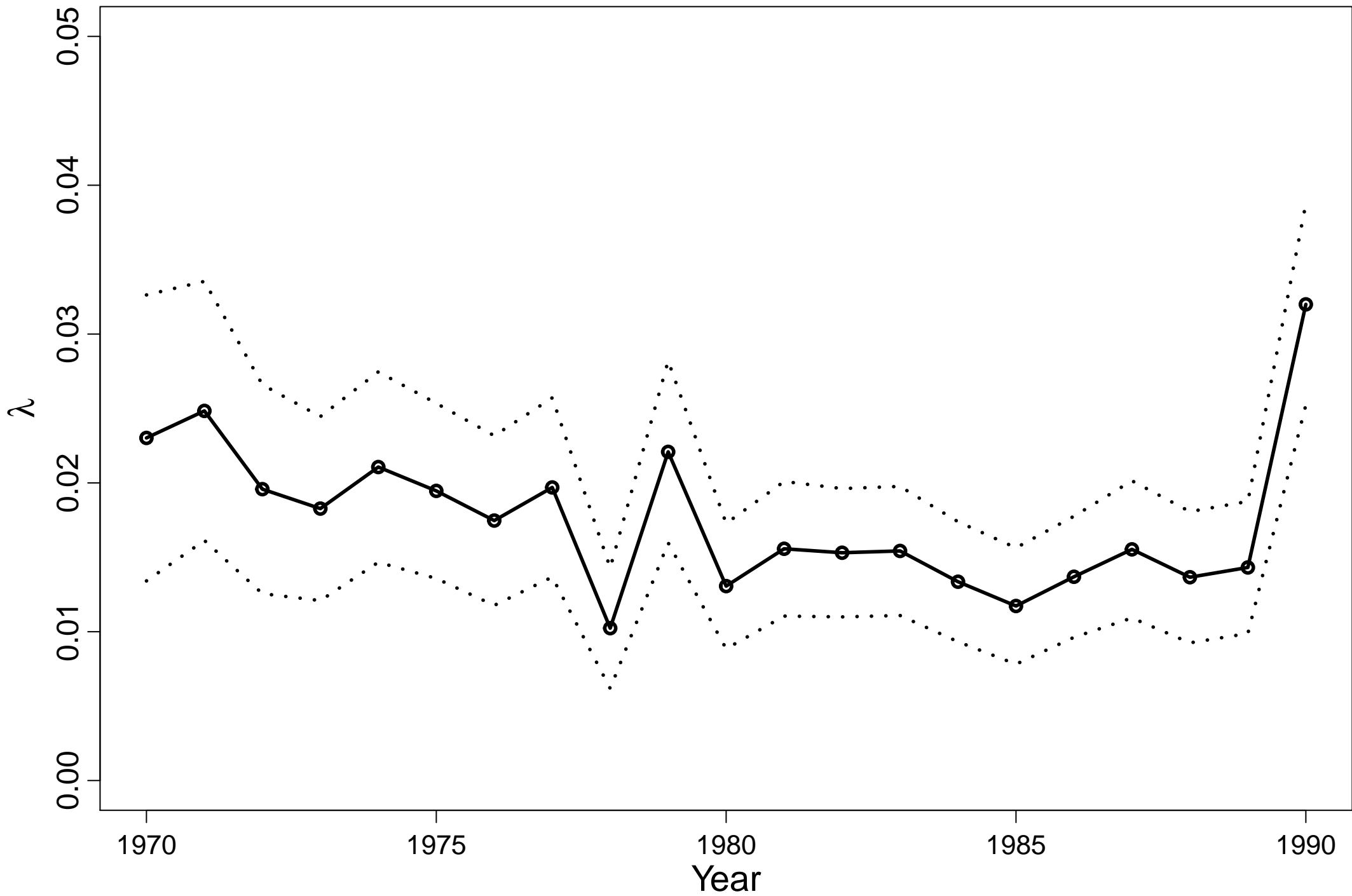

Supplement: Supplementary file 2 [file ECE3-9-769-s002.pdf]

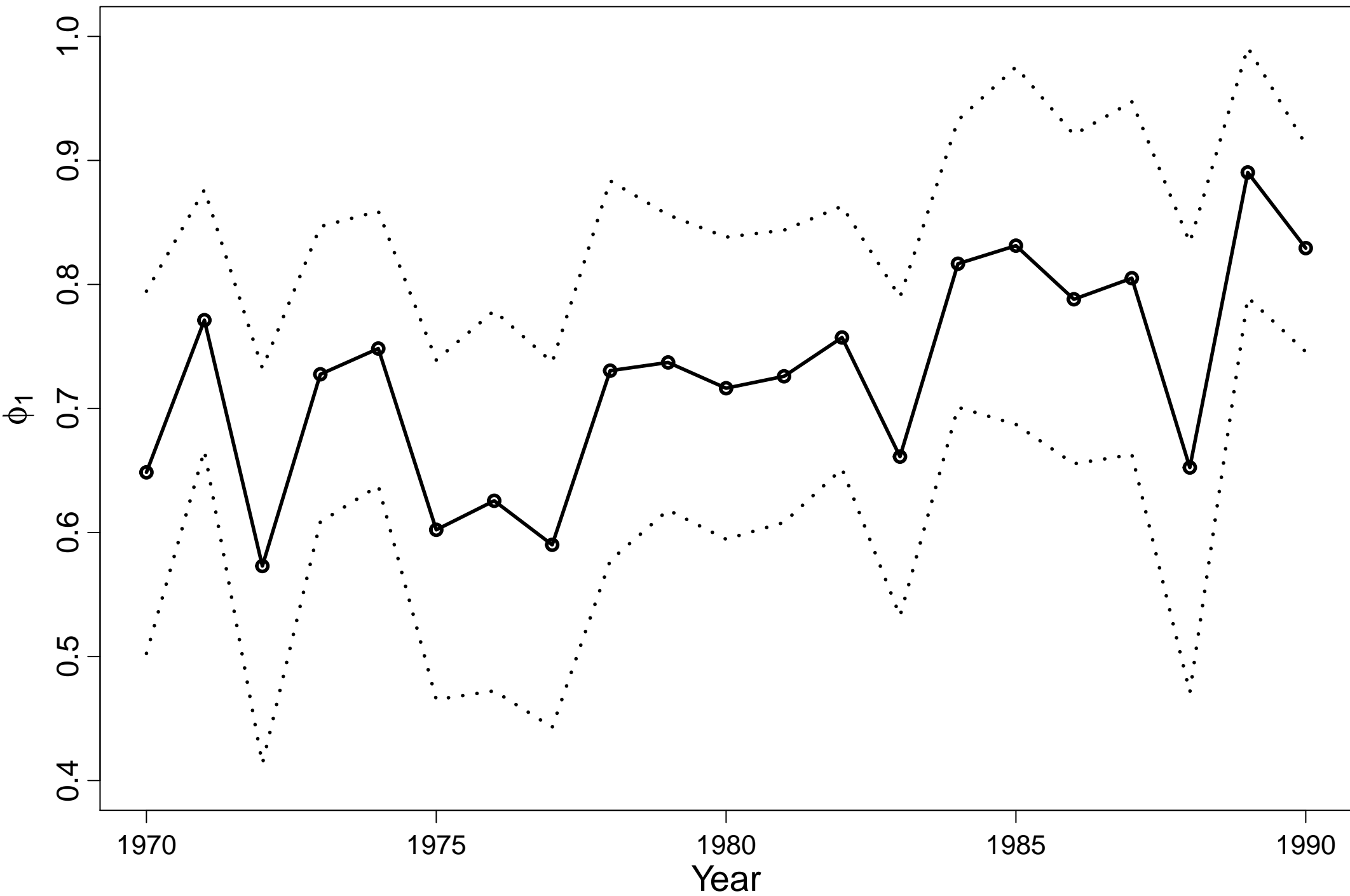

Supplement: Supplementary file 3 [file ECE3-9-769-s003.pdf]
